# Glycosylated NS3/NS3A protein of bluetongue virus facilitates efficient viral egress via lipid raft anchoring

**DOI:** 10.1128/jvi.02144-25

**Published:** 2026-02-18

**Authors:** Yingran Huang, Yinglin Qi, Junyong Guan, Ran Shao, Cankun Xi, Xing Liu, Dong Zhou, Shuhui Qi, Xin Yin

**Affiliations:** 1 State Key Laboratory of Animal Disease Control and Prevention, Harbin Veterinary Research Institute, Chinese Academy of Agricultural Sciences 687216 , Harbin, China; University of Michigan Medical School, Ann Arbor, Michigan, USA

**Keywords:** bluetongue virus, NS3/NS3A, N-linked glycosylation, membrane trafficking, lipid rafts, viral egress, pathogenesis

## Abstract

Bluetongue virus (BTV) is an economically important arbovirus of ruminants worldwide. The nonstructural glycoprotein NS3/NS3A contains a uniquely conserved N-linked glycosylation site at Asn_150_; yet, the functional consequences of this modification have remained unclear. Here, we characterize the Asn_150_-linked glycan and show that it predominantly comprises high-mannose structures that undergo maturation specifically in mammalian cells. Using a reverse-genetics system, we demonstrate that N-linked glycosylation is not required to maintain NS3/NS3A stability or its interaction with outer capsid proteins, but is essential for efficient virion egress. Ablation of this site (N150Q) markedly impaired plasma-membrane localization and reduced partitioning into detergent-resistant lipid raft microdomains. These defects resulted in reduced extracellular titers and attenuated multicycle spread. Together, these findings support a model in which the Asn_150_ glycan functions as a glycan-dependent targeting signal that biases NS3/NS3A toward plasma-membrane microdomains enriched in lipid rafts, thereby promoting productive virus release. In AG129 mice, the N150Q mutant exhibited attenuated virulence, characterized by reduced viremia, restricted tissue dissemination, and minimal pathology, in contrast to the severe systemic disease caused by the wild-type virus. The strict conservation of the Asn_150_ motif across BTV serotypes underscores its functional importance and highlights NS3/NS3A glycosylation as a potential target for broad-spectrum antivirals or vaccine strategies.

**IMPORTANCE:**

Bluetongue virus (BTV) is an economically significant arbovirus that causes hemorrhagic disease in ruminants and restricts global livestock trade. Although the N-linked glycosylation site at Asn_150_ in the nonstructural protein NS3/NS3A is strictly conserved across BTV serotypes, its functional role has remained unclear. Here, we demonstrate that glycosylation at Asn_150_ acts as a targeting signal that promotes efficient trafficking of NS3/NS3A to the plasma membrane and its enrichment within lipid raft-associated microdomains that support virus egress. These findings reveal that this viral glycan plays a critical role in directing NS3/NS3A to specific membrane microenvironments, providing new insight into how BTV exploits host membrane organization to facilitate its replication cycle. The strict conservation of this glycosylation site among typical BTV serotypes highlights its potential as a broad-spectrum antiviral target and warrants further investigation into the role of NS3/NS3A glycosylation in vaccine development and interspecies transmission.

## INTRODUCTION

Bluetongue disease represents a significant threat to both domestic and wild ruminants, with mortality rates exceeding 70% in susceptible sheep populations (1) and annual agricultural losses estimated at over $3 billion globally (2). The causative agent, bluetongue virus (BTV), belongs to the genus *Orbivirus* within the family *Sedoreoviridae*. Due to its significant epidemic potential, it is classified as a notifiable disease agent by the World Organization for Animal Health (WOAH) (1, 3–7). The 2023 emergence of a novel serotype 3 strain in the Netherlands, which spread to nearly 6,000 farms within 2 months, underscores the ongoing threat of re-emergence (8). Reports of infection in atypical hosts, including fatal disease and abortion in dogs, further suggest an expanding host range and increasing opportunities for cross-species transmission (9). These epidemiological features, combined with the virus’s high mutation rate and persistent challenges in containment, highlight the urgent need to elucidate molecular determinants of BTV replication and pathogenesis to guide rational vaccine and antiviral development (8, 10, 11).

The BTV genome comprises ten double-stranded RNA segments that encode seven structural proteins (VP1–VP7) and five nonstructural proteins (NS1, NS2, NS3/NS3A, NS4, and NS5) (12–14). Among these, the outer capsid proteins VP2 and VP5 drive serotype specificity (15–18) and contribute to virulence, making them primary targets for conventional vaccines (19). However, accumulating evidence identifies NS3/NS3A as a critical virulence determinant (6, 19–23). Its strict evolutionary conservation across serotypes demonstrated the role in pathogenicity position NS3/NS3A as a promising candidate for pan-serotype intervention strategies (6, 19–23), underscoring the imperative to define its molecular functions in viral pathogenesis. NS3/NS3A is encoded by segment 10, in which NS3A is translated from the second in-frame AUG codon and lacks the first 13 amino acids present in NS3 (24). Roy et al. and others have conducted extensive and pioneering work demonstrating that this membrane-associated glycoprotein orchestrates multiple stages of viral propagation by facilitating viral maturation (25), intracellular trafficking of virus-like particles (VLP) (26–29), virion egress through vesicle-associated pathways (30–32), and host immune responses (33–35). Notably, NS3/NS3A contains the only functional N-linked glycosylation motif encoded by the BTV genome (36). Although previous structural and heterologous expression studies have suggested that glycosylation of NS3/NS3A may regulate its subcellular localization (36–38), its biological significance during natural infection has remained unresolved.

N-linked glycosylation is the most common form of protein glycosylation, involving the attachment of an oligosaccharide to an asparagine residue within the consensus sequence Asn-X-Ser/Thr, where X represents any amino acid except proline (39). This process is initiated co-translationally in the endoplasmic reticulum (ER) and continues along the secretory pathway (40). As a ubiquitous host-derived post-translational modification, N-linked glycosylation is widely exploited by viruses to enhance infectivity (41, 42). In enveloped viruses such as human immunodeficiency virus-1 (HIV-1), Japanese encephalitis virus (JEV), and West Nile virus (WNV), glycosylation of the envelope protein facilitates receptor binding and membrane fusion (43–45). In SARS-CoV-2, spike glycosylation modulates antigenicity, masks neutralizing epitopes, and shapes conformational dynamics, thereby promoting immune evasion (46–48). Beyond immune modulation, glycosylation contributes broadly to protein folding, stability, trafficking, and expression efficiency (48–52). Thus, defining how viral proteins utilize host glycosylation pathways is central to understanding host-pathogen interactions and informing vaccine and therapeutic design.

In this study, we investigated the biological significance of the conserved N-linked glycosylation motif in NS3/NS3A during authentic BTV infection. By integrating glycan profiling, reverse genetics, interactome analysis, and *in vivo* validation, we addressed whether this modification confers a selective advantage during infection or represents a nonessential evolutionary remnant. Our results establish a mechanistic framework in which NS3/NS3A glycosylation facilitates the efficient trafficking of NS3/NS3A to the plasma membrane and promotes its enrichment within lipid raft-associated microdomains. This process enhances virion egress and systemic dissemination, providing valuable insights for the development of next-generation antivirals and vaccines.

## MATERIALS AND METHODS

### Cells and cell culture

BHK-21 (baby hamster kidney cells; ATCC CCL-10), BHK-T7/5 (BHK-21 derivative stably expressing T7 RNA polymerase), HEK-293T (human embryonic kidney cells; ATCC CRL-3216), HeLa (ATCC CCL-2), and MDOK (ovine kidney cells; ATCC CRL-1633) were cultured in custom Dulbecco’s modified Eagle’s medium (DMEM) supplemented with 10% heat-inactivated fetal bovine serum (FBS), 100 U/mL penicillin, and 100 μg/mL streptomycin. Cells were maintained at 37°C in a humidified incubator with 5% CO₂. C6/36 cells (Aedes albopictus clone; ATCC CRL-1660) were maintained in custom Eagle’s minimum essential medium supplemented with 10% heat-inactivated FBS, 100 U/mL penicillin, and 100 μg/mL streptomycin at 28°C in a humidified incubator with 5% CO₂.

### Experimental time points

Time points were determined based on comparable stages of the viral replication cycle rather than fixed hours post-infection or post-transfection, to account for differences in replication kinetics and the onset of cytopathic effects across cell lines. For high multiplicity of infection (MOI) infections (MOI = 5), samples were collected at 0, 3, 6, and 12 h post-infection (hpi) in HEK-293T cells and at 0, 6, 12, and 18 hpi in MDOK cells. Under low MOI conditions (MOI = 0.01), sampling was performed at 6, 12, 18, and 24 hpi in HEK-293T cells and at 12, 18, 24, and 30 hpi in MDOK cells. For transmission electron microscopy (TEM) and pharmacological assays, a single time point of 12 hpi was used. In transfection experiments, HEK-293T cells were harvested at 20 h post-transfection (hpt). For cycloheximide (CHX) chase assays, cells were treated with 100 µg/mL CHX at 10 hpi or 18 hpt, and samples were collected at 4-h intervals thereafter. The rationale for each selected time point, corresponding to distinct phases of viral replication and protein accumulation, is described in the relevant sections of the manuscript.

### Mice

AG129 mice, which lack both type I and type II interferon receptors, were generously provided by Prof. Jian-ping Zuo (Institut Pasteur of Shanghai, Chinese Academy of Sciences). Mice were bred and maintained under specific pathogen-free (SPF) conditions at the Harbin Veterinary Research Institute (HVRI), Chinese Academy of Agricultural Sciences (CAAS).

### Antibodies

The following antibodies were generated in-house: mouse monoclonal anti-BTV VP7, and mouse and rabbit polyclonal sera against BTV NS3/NS3A. Commercial antibodies used in this study included anti-FLAG (DYKDDDDK) monoclonal antibody (1:1,000 for immunofluorescence assay [IFA], 1:10,000 for western blotting [WB]; 66008-4-Ig, Proteintech), anti-HA polyclonal antibody (1:100 for IFA, 1:1,000 for WB; 51064-2-AP, Proteintech), anti-β-actin monoclonal antibody (1:10,000 for WB; 66009-1-Ig, Proteintech), anti-Calnexin polyclonal antibody (1:200 for IFA; 10427-2-AP, Proteintech), anti-Syntaxin 6 polyclonal antibody (1:200 for IFA; 10841-1-AP, Proteintech), anti-Flotillin 1 monoclonal antibody (1:100 for IFA; 67968-1-Ig, Proteintech), and anti-Filamin A (FLNA) monoclonal antibody (1:1,000 for WB; 67133-1-Ig, Proteintech). Secondary antibodies included Alexa Fluor 488- or 568-conjugated goat anti-mouse/rabbit IgG (H+L) highly cross-adsorbed antibodies (1:1,000; Invitrogen), Wheat Germ Agglutinin Alexa Fluor Plus 488 (1:1000; W11261, Invitrogen), and HRP-conjugated goat anti-rabbit and anti-mouse IgG (1:5,000 for WB; 31,460 and 62-6520, Thermo Fisher).

### Plasmids and transfection

Plasmids were constructed using standard molecular cloning techniques. Site-directed mutagenesis was performed by overlapping PCR using PrimeSTAR HS DNA Polymerase (Takara Bio, R010A), according to the manufacturer’s protocol. All plasmids used in this study are listed in Table S2.

For transfection, HEK-293T cells (1.2 × 10⁶ cells/well in six-well plates) were transfected with the indicated plasmids using Lipofectamine 3000 (Thermo Fisher Scientific). Samples were collected 20 h post-transfection for downstream analyses.

### Immunofluorescence assay

Cells were washed with PBS, fixed with 4% paraformaldehyde (15 min, RT), and permeabilized with 0.01% Triton X-100. After blocking with 1% BSA, cells were incubated with primary antibodies overnight at 4°C. After washing with PBS, cells were incubated with secondary antibodies for 45 min at 37°C, then stained with DAPI for 15 min at room temperature. Images were acquired using EVOS M5000 inverted fluorescence microscopy or Airyscan LSM800 confocal microscopy.

### Virus production, infection, and titration

The NS3/NS3A glycosylation-defective mutant virus (BTV _N150Q_) was generated by introducing an N150Q substitution into segment 10 of the BTV-20 GX015/China/2013 strain and the BTV-1 South Africa strain using plasmid-based reverse genetics, as previously described (53–55). Virus stocks were propagated by infecting BSR cells and titrated using the tissue culture infectious dose 50 (TCID₅₀/mL) assay. For time-course infection experiments, BTV strains were propagated in MDOK and HEK-293T cells. Cell monolayers were infected separately with BTV _WT_ or BTV _N150Q_ at an MOI of 0.01 or 5. After 1–1.5 h of incubation at 37°C, cells were washed three times with PBS and maintained in DMEM supplemented with 2% FBS. Time points were selected to represent comparable stages of the viral replication cycle in each cell line, accounting for differences in replication kinetics and the onset of cytopathic effects, rather than using identical hours post-infection. For viral titration, virus-containing supernatants were serially diluted (10^−^¹ to 10^−^¹²) in DMEM supplemented with 2% FBS and inoculated onto BSR cell monolayers in 96-well plates. After 7 days of incubation, viral titers were calculated using the Spearman-Kärber method and expressed as TCID₅₀ per milliliter.

### qRT-PCR assay for viral RNA

Total RNA was extracted using either TRIzol reagent or the SimplyP Total RNA Extraction Kit (Bioflux, China). Quantitative RT-PCR was performed using the HiScript II U+ One Step qRT-PCR Probe Kit (Vazyme, China) on a QuantStudio 5 Real-Time PCR System (Applied Biosystems, USA). Viral RNA levels were quantified against a synthetic RNA standard. Primer and probe sequences are listed in Table S3.

### Co-immunoprecipitation assay

To assess interactions between NS3/NS3A (WT or N150Q) and the outer capsid proteins VP2 and VP5, HEK-293T cells were co-transfected with pCAGGS-NS3/NS3A_BTV-20/WT_ or pCAGGS-NS3/NS3A_BTV-20/N150Q_ together with pCAGGS-VP2_BTV-20_-2×HA or pCAGGS-VP5_BTV-20_-2×HA, as indicated. At 20 h post-transfection, cells were washed three times with phosphate-buffered saline (PBS) and lysed on ice for 30 min in lysis buffer containing 150 mM NaCl, 50 mM Tris-HCl (pH 8.0), 0.8% NP-40, and 100 μM phenylmethylsulfonyl fluoride (PMSF).

Cell lysates were clarified by centrifugation at 12,000 × *g* for 15 min at 4°C, and the supernatants were incubated with 30 μL of anti-HA magnetic beads (HY-K0201-1, MedChemExpress) overnight at 4°C with gentle rotation. Beads were washed five times with lysis buffer, and bound proteins were eluted by boiling in SDS sample loading buffer for 10 min at 100°C. Eluted proteins were analyzed by SDS-PAGE followed by immunoblotting with the indicated antibodies.

### Affinity selection mass spectrometry and glycoprofile analysis of BTV NS3/NS3A

NS3/NS3A _WT_ or the glycosylation-deficient mutant NS3/NS3A _N150Q_ was expressed in HEK-293T cells by transient transfection with pCAGGS-NS3/NS3A_BTV-20/WT_-3×FLAG-2×HA or pCAGGS-NS3/NS3A_BTV-20/N150Q_-3×FLAG-2×HA plasmids. Cells were harvested at 20 h post-transfection and washed twice with ice-cold PBS. Cell pellets were lysed on ice in lysis buffer (150 mM NaCl, 50 mM Tris-HCl, pH 7.5, 0.8% NP-40) supplemented with PMSF and a protease inhibitor cocktail. Lysates were clarified by centrifugation at 12,000 × *g* for 15 min at 4°C, and the supernatants were incubated with anti-FLAG antibody-conjugated beads (Proteintech, 66008-4-Ig) for 12 h at 4°C with gentle rotation.

Following incubation, beads were washed extensively with lysis buffer to remove nonspecifically bound proteins. Bound NS3/NS3A proteins were eluted by incubation with 3×FLAG peptide (150–200 μg/mL) in elution buffer (50 mM Tris-HCl, pH 7.5, 150 mM NaCl) for 30 min at 4°C. Eluted fractions were collected and analyzed by SDS-PAGE followed by Coomassie blue staining to assess protein purity and integrity. Purified NS3/NS3A proteins were subsequently used as input material for affinity-based mass spectrometry (AS-MS) to analyze associated host factors or subjected to glycoprofile characterization.

### Plasma membrane separation assay

HEK-293T cells were seeded in six-well plates and transfected with NS3/NS3A plasmids. Plasma membrane and cytosolic fractions were isolated using the Minute Plasma Membrane Protein Isolation and Cell Fractionation Kit (Invent Biotechnologies) (56, 57), following the manufacturer’s instructions. Samples were analyzed by Western blot.

### Raft membrane preparation

Raft membranes were isolated according to previously established protocols (58, 59). Briefly, samples were lysed with TNET buffer (20 mM Tris-HCl, pH 8.0, 150 mM NaCl, 1 mM EDTA, and 1% Triton X-100) while sucrose gradients were prepared using TNE buffer (without Triton X-100) supplemented with a protease inhibitor cocktail. Transfected 293T or BTV-infected MDOK cells grown in 15 cm dishes were washed with cold PBS, scraped into 0.5 mL of cold TNET buffer, and incubated on ice for 30 min. Lysates were sheared by passing through a 23-gauge needle 10 times, adjusted to 40% sucrose or OptiPrep, and overlaid with 35% and 5% sucrose gradients. Samples were centrifuged at 180,000 × *g* for 18 h at 4°C using a Beckman SW41 rotor. Fractions were collected from the top of the gradient.

### CHX chase assays

MDOK cells were infected at an MOI = 10, and HEK-293T cells were transfected with expression plasmids using Lipofectamine 3000. At 10 h post-infection or 18 h post-transfection, cells were treated with 100 μg/mL CHX. Cells were harvested every 4 h after CHX treatment, lysed in 100 μL lysis buffer, and analyzed by western blot to monitor protein degradation.

### Pharmaceutical treatments

For infection experiments, cells were treated with 5 mM methyl-β-cyclodextrin (MβCD; Sigma-Aldrich, C4555) at 6 h post-infection (h.p.i.) for 1 h, followed by three washings with PBS. Cells were then maintained in DMEM, and viral titers were determined at 12 hpi. For transfection experiments, cells were treated with 5 mM MβCD for 30 min, followed by PBS washing and subsequently processed for confocal microscopy. Alternatively, cells were treated with 5 μM trifluoperazine (TFP; Aladdin, C118530-5g). For confocal microscopy, TFP was added at 20 h post-transfection for 10 min. For infection experiments, TFP was added at 6 hpi and maintained for 6 h, and cells were harvested at 12 hpi for viral titration.

### LDH release assay

Cells were seeded into 96-well plates at a density of 6 × 10^4^ cells per well. Cells were treated with increasing concentrations of methyl-β-cyclodextrin (MβCD; 0, 5, 10, and 15 mM) for 1 h, followed by PBS washes prior to LDH detection, or with trifluoperazine (TFP; 0, 5, 10, and 15 μM) for 6 h. LDH release was measured using the LDH Cytotoxicity Detection Kit (Beyotime, C0016) according to the manufacturer’s instructions. Absorbance was measured at 490 nm, and LDH activity was expressed as optical density (OD₄₉₀).

### Assessment of plasma membrane integrity by trypan blue exclusion

Plasma membrane integrity was assessed using the trypan blue exclusion assay. Cell suspensions were mixed 1:1 (vol/vol) with 0.4% trypan blue solution (Invitrogen) and incubated for 3 min at room temperature (RT). Subsequently, 10 μL of the mixture was loaded onto a Countess II FL chamber slide (Invitrogen). Viable and non-viable cells were quantified using the Countess II FL Automated Cell Counter (Invitrogen).

### Western blot

For detection of released proteins, culture supernatants were centrifuged at 1,000 × *g* for 10 min to remove cellular debris, then concentrated approximately 10-fold using a 10 kDa ultrafiltration membrane (Millipore). The concentrates were mixed with 5× SDS reducing sample buffer to a final concentration of 1× and boiled at 100°C for 15 min. For cell lysis, cells were incubated on ice for 10 min in lysis buffer containing PMSF, followed by centrifugation at 12,000 × *g* for 10 min at 4°C. The resulting supernatant was mixed with 5× SDS reducing sample buffer to a final concentration of 1× and boiled at 100°C for 15 min.

Proteins were separated by SDS-PAGE and transferred to PVDF membranes (Millipore). After blocking with 5% (wt/vol) bovine serum albumin (BSA) in TBST, membranes were incubated overnight at 4°C with the appropriate primary antibodies, followed by incubation with secondary antibodies at room temperature for 1 h. Membranes were scanned using the TOUCH IMAGER system (e-BLOT), and specific protein bands were quantified using ImageJ software.

## RESULTS

### Conservation of the Asn_150_ glycosylation site in NS3/NS3A across BTV serotypes

**Fig 1:**
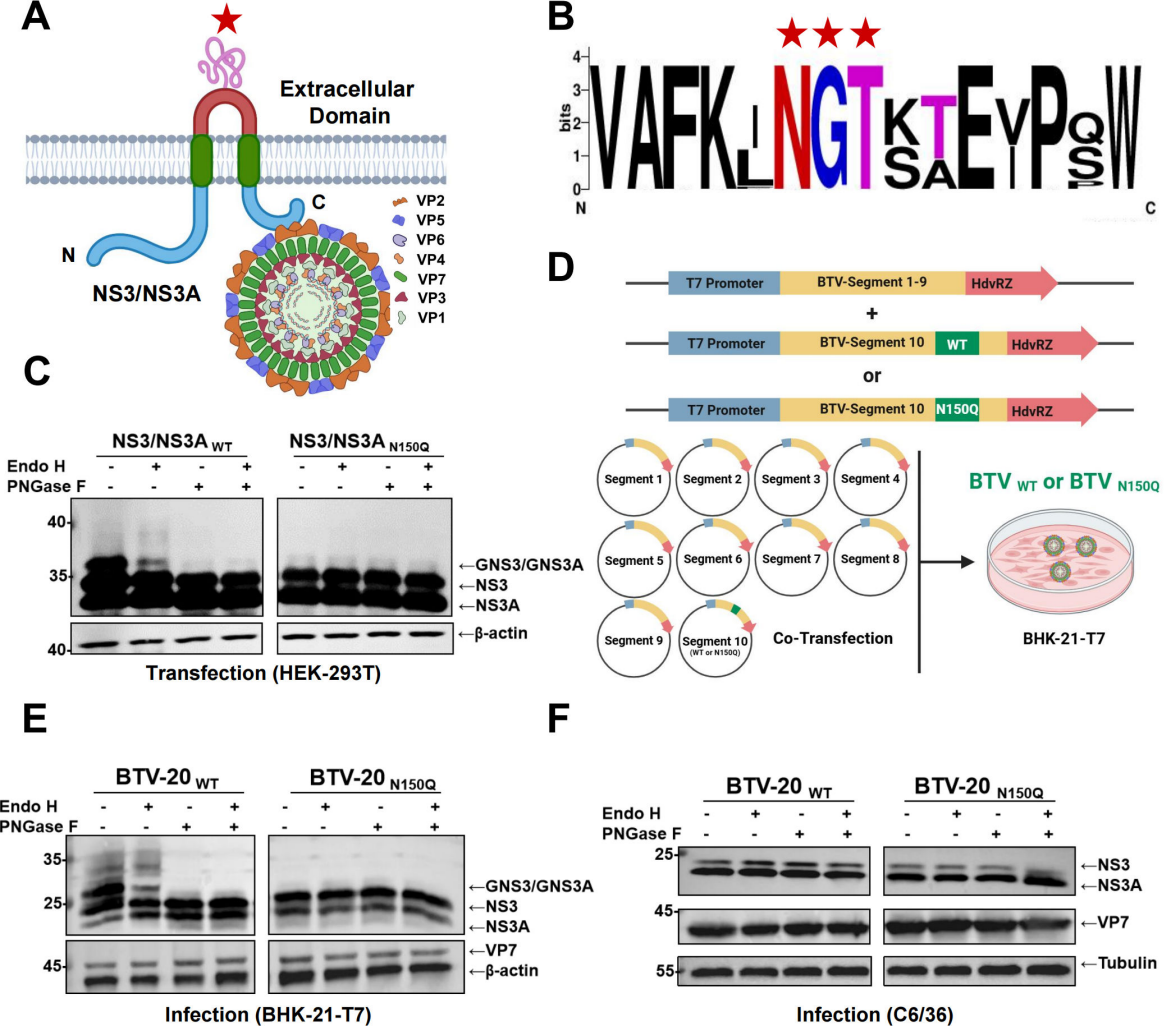
The conservation of the Asn_150_ glycosylation site in NS3/NS3A across Bluetongue virus (BTV) serotypes. (**A**) Predicted membrane topology of BTV NS3/NS3A. The extracellular domain containing the N150 glycosylation motif (red star) is flanked by transmembrane helices (green boxes). The C-terminal domain of NS3 interacts with the outer capsid proteins VP5 and VP2 of BTV. Schematic illustrations were generated using BioRender (https://www.biorender.com/library). (**B**) Conservation analysis of the Asn-X-Ser/Thr motif (red stars) across 24 typical BTV serotypes. (**C**) Glycosidase sensitivity of NS3/NS3A constructs (wild-type or N150Q mutant) in transfected HEK-293T cells. Cell lysates harvested at 20 h post-transfection were treated with PNGase F or Endo H and analyzed by immunoblotting with anti-NS3/NS3A serum. (**D**) Schematic diagram of the reverse genetics system used to generate recombinant BTV. The NS3/NS3A glycosylation-defective mutant virus (BTV _N150Q_) was generated by introducing the N150Q substitution into segment 10 of BTV-20 or BTV-1. (**E**) Validation of NS3/NS3A glycosylation in BTV-20-infected BHK-21-T7 cells (MOI = 10, 24 h post-infection). Cell lysates from WT and N150Q virus-infected cells were treated with PNGase F or Endo H and analyzed by immunoblotting. (**F**) Validation of NS3/NS3A glycosylation in BTV-20-infected C6/36 cells (MOI = 10, 96 h post-infection). Lysates were treated with PNGase F or Endo H and subjected to immunoblotting.

The topology of the BTV NS3/NS3A protein consists of intracellular N- and C-terminal domains flanking two transmembrane helices and a conserved extracellular loop (36). The C-terminal domain of NS3/NS3A interacts with the outer capsid proteins VP5 and VP2 of BTV (Fig. 1A) (31, 37). Notably, a single N-glycosylation site that gives rise to glycans is present in the extracellular region (36, 37). Systematic sequence alignment of 24 typical BTV serotypes revealed strict conservation of a single N-linked glycosylation motif Asn-X-Ser/Thr (where X represents any amino acid except proline) (Fig. 1B and Table S1). We next characterized the glycan profile of NS3/NS3A purified from HEK-293T cells and found that it is predominantly modified with a heterogeneous population of oligomannose-type N-glycans, ranging from HexNAc₂Hex₁ to HexNAc₂Hex₁₂ (Fig. S1A and S1B). Consistent with this, treatment with PNGase F, which removes all N-linked glycans, converted the diffuse, higher-molecular-weight NS3/NS3A species into two lower bands corresponding to unglycosylated NS3 and NS3A (Fig. 1C). In contrast, Endo H treatment shifted only a subset of glycosylated NS3/NS3A species. Notably, PNGase F treatment had no effect on the electrophoretic mobility of the NS3/NS3A_N150Q_ mutant, indicating the absence of N-linked glycosylation. Together, these results confirm that N150 is the sole functional N-linked glycosylation site in NS3/NS3A and demonstrate that NS3/NS3A acquires a heterogeneous pool of oligomannose-type glycans. To further examine the functional relevance of NS3/NS3A glycosylation, both BTV-1 and BTV-20 were selected as representatives of distinct serotypes. The wild-type (WT) and NS3/NS3A glycosylation-deficient viruses were rescued using a plasmid-based reverse genetics system (Fig. 1D) (53). The glycosylation pattern of infection-derived NS3/NS3A in mammalian cells closely resembled that observed under transfection conditions, showing complete sensitivity to PNGase F and partial sensitivity to Endo H (Fig. 1E; Fig. S1C). This agreement between the two expression systems confirms that the N150Q mutation effectively abolishes N-linked glycosylation irrespective of experimental context. A clear host-specific difference emerged when comparing NS3/NS3A expressed in mammalian cells with that produced in mosquito (C6/36) cells (Fig. 1F). Mammalian-derived NS3/NS3A displayed a prominent electrophoretic shift following PNGase F digestion, indicative of robust glycosylation. In contrast, NS3/NS3A expressed in C6/36 cells exhibited minimal mobility changes upon PNGase F treatment, suggesting substantially reduced or altered glycosylation in the arthropod system. Taken together, these results demonstrate that Asn_150_ serves as the functional N-glycosylation site in NS3/NS3A across representative BTV serotypes and that the extent of glycan maturation is strongly influenced by the host cell type.

### NS3/NS3A N-linked glycosylation at residue N150 is required for efficient infectious viral particle release

**Fig 2:**
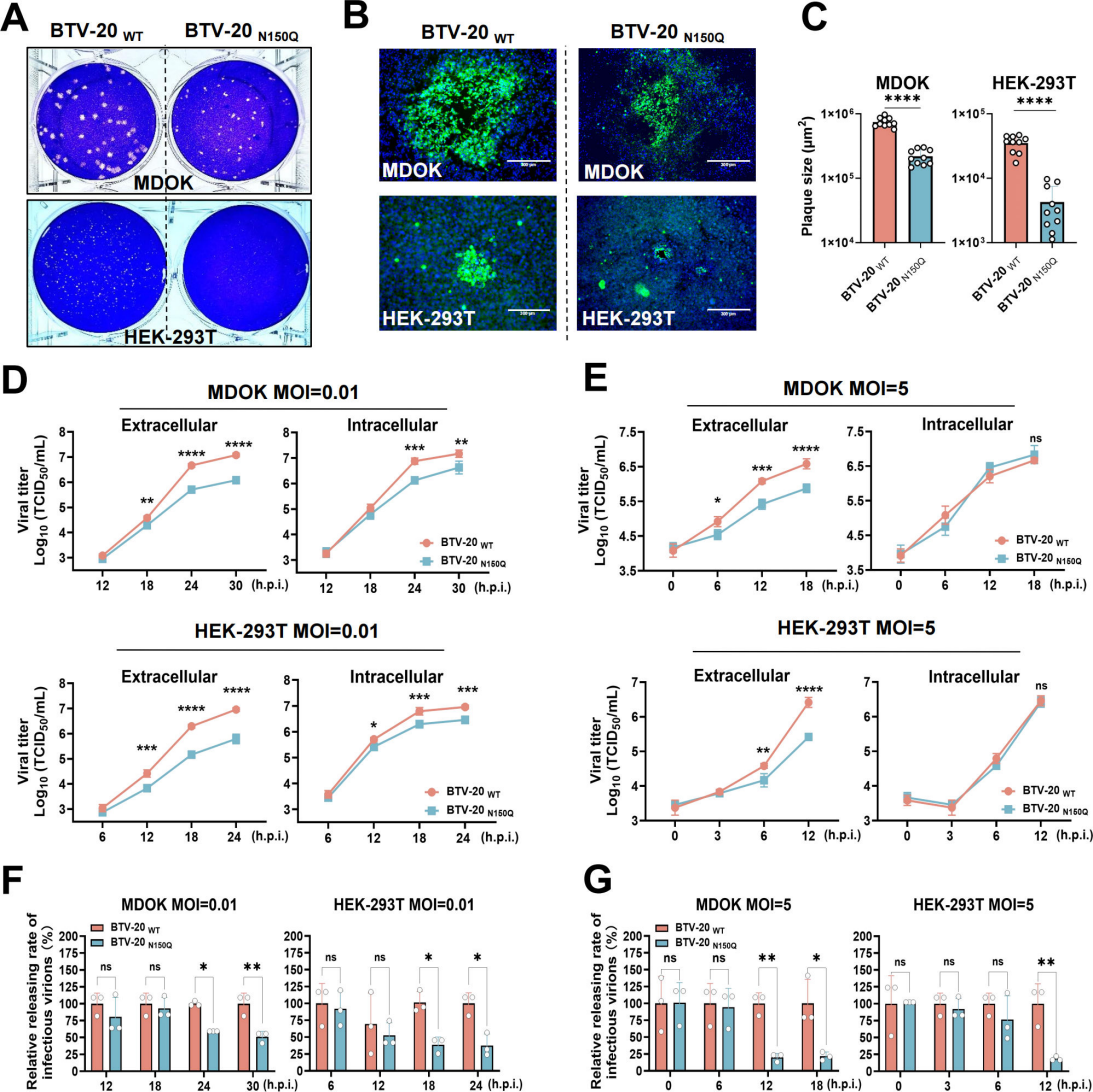
Loss of N-linked glycosylation of NS3/NS3A reduces BTV-20 infective virions release in mammalian cells. (**A**) Plaques in MDOK and HEK-293T cells infected with either BTV-20 _WT_ or BTV-20 _N150Q_. Monolayers were infected with BTV-20 _WT_ and BTV-20 _N150Q_ and overlaid with low-melting-point agarose. Plaques were visualized by crystal violet staining at 7 d.p.i. (MDOK) or 3 d.p.i. (HEK-293T). (**B**) Immunofluorescence-based plaque assay. Infected cells were overlaid with methylcellulose, fixed at 7 d.p.i. (MDOK) or 5 d.p.i. (HEK-293T), and stained with an anti-VP7 monoclonal antibody followed by an Alexa Fluor 488-conjugated secondary antibody. Scale bar, 300 μm. (**C**) Quantification of plaque areas (*n* = 10 plaques per virus) from panel B using ImageJ. Data are presented as mean ± SD (two-tailed unpaired t-test; ****P < 0.0001). (D–E) Multicycle (MOI = 0.01; **D**) and single-cycle (MOI = 5; **E**) growth kinetics of BTV-20 _WT_ and BTV-20 _N150Q_ in MDOK and HEK-293T cells. Extracellular and intracellular viral titers were quantified by TCID₅₀ assay. Data were log₁₀-transformed and are presented as mean ± SD (*n* = 3 biological replicates; two-way ANOVA with Šídák’s multiple-comparison test; *P < 0.05, **P < 0.01, ***P < 0.001, ****P < 0.0001). (**F and G**) Relative releasing efficiency was calculated as [extracellular/(extracellular + intracellular)] for each condition. Bars represent mean ± SD (*n* = 3 biological replicates; ns, not significant; *P < 0.05, **P < 0.01, ***P < 0.001, ****P < 0.0001; two-way ANOVA with Šídák’s test).

To investigate the functional role of N-linked glycosylation of NS3/NS3A during BTV infection, we compared the replication characteristics of WT BTV-20 and BTV-1 with their corresponding N150Q mutants in MDOK and HEK-293T cells. Plaque assays revealed that both BTV-20 _N150Q_ and BTV-1 _N150Q_ formed significantly smaller plaques than their respective parental viruses in both cell types (Fig. 2A; Fig. S2A), indicating that loss of glycosylation at Asn_150_ impairs viral spread in cell culture. Immunofluorescence analysis further showed that the N150Q mutations caused a marked reduction in foci size compared with WT viruses (Fig. 2B and C; Fig. S2B and C). Consistent with these observations, multicycle growth kinetics demonstrated that infectious titers of BTV-20_N150Q_ and BTV-1_N150Q_ were consistently lower than those of their respective WT viruses (Fig. 2D; Fig. S2D). In single-cycle growth experiments, both N150Q mutants exhibited markedly reduced extracellular viral titers at 12 h post-infection (h.p.i.), while intracellular titers remained comparable to WT (Fig. 2E; Fig. S2E). Analysis of virus release efficiency further revealed that loss of NS3/NS3A glycosylation impaired the release of infectious virions (Fig. 2F and G; Fig. S2F and G). Together, these findings indicate that N-linked glycosylation at Asn_150_ promotes efficient BTV propagation by facilitating late-stage events associated with virion egress.

### The glycosylation of NS3/NS3A enhanced the release of BTV outer capsid proteins

**Fig 3:**
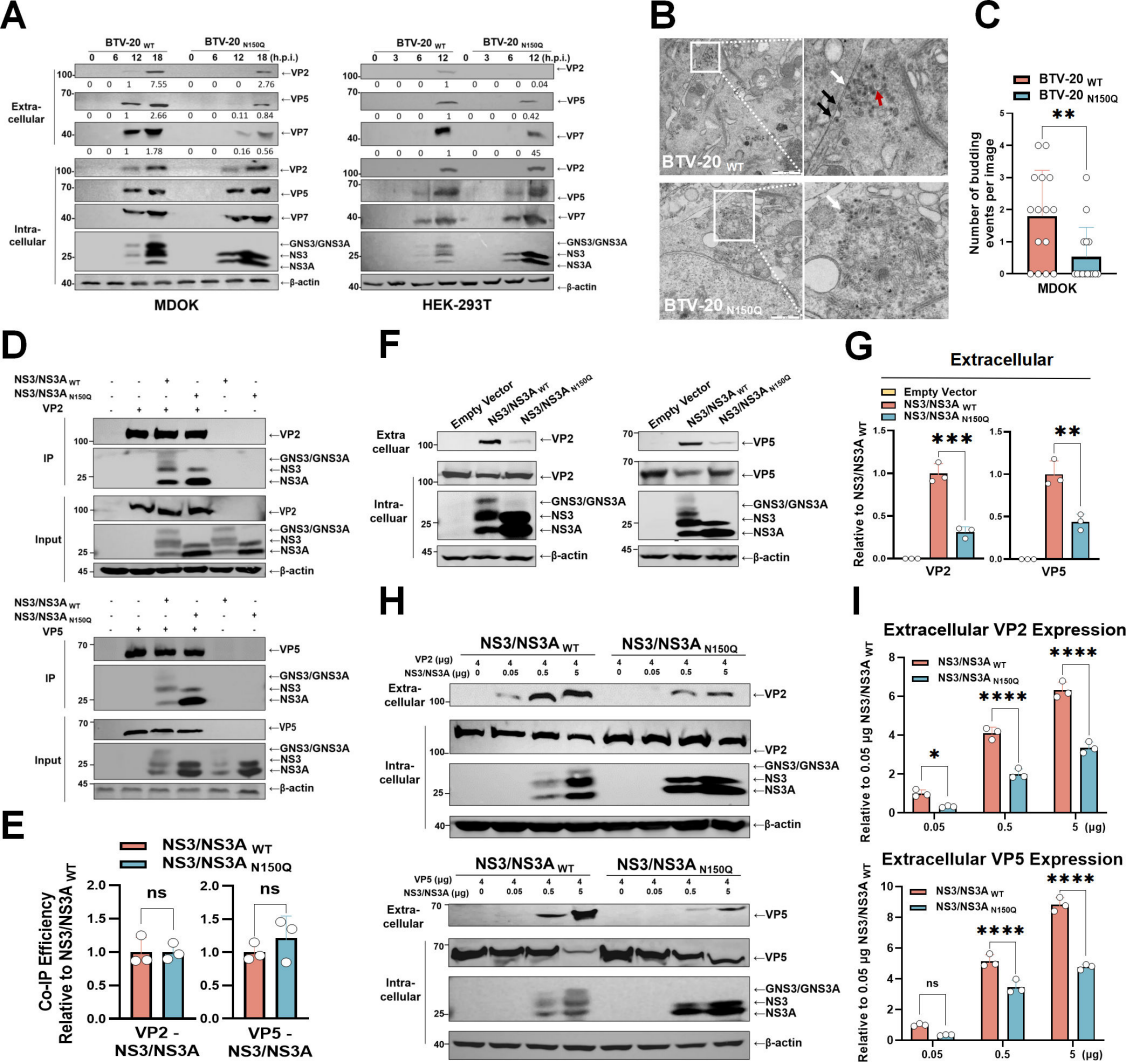
N-linked glycosylation facilitates NS3/NS3A-mediated capsid protein trafficking and virion egress. (**A**) Time-course expression of viral proteins in MDOK and HEK-293T cells infected with BTV-20 _WT_ or BTV-20 _N150Q_ (MOI = 10). Cells were harvested at the indicated time points and analyzed by Western blot. Densitometric values of extracellular VP2 and VP5 bands (normalized using ImageJ) are shown beneath each blot. (**B**) Representative TEM images of MDOK cells infected with BTV-20 _WT_ or BTV-20 _N150Q_ (MOI = 10; 12 h.p.i.). White arrows indicate membrane curvature consistent with budding profiles; black arrows indicate virions adjacent to the plasma membrane; the red arrow indicates membrane-wrapped virions. Scale bar: 1 μm. Magnified regions corresponding to the boxed areas are shown on the right. (**C**) Quantification of budding events in TEM images. Each dot represents the number of budding profiles counted in a single micrograph (*n* = 15 independent images per virus). Bars show mean ± SD (two-tailed unpaired t-test; **P < 0.01). (**D**) Co-immunoprecipitation (Co-IP) of VP2 or VP5 with NS3/NS3A variants. Lysates from HEK-293T cells co-transfected with VP2 or VP5 along with NS3/NS3A (WT or N150Q) expression plasmids were immunoprecipitated. (**E**) Quantification of Co-IP efficiency from panel D. Data represent mean ± SD (ns, not significant; two-tailed unpaired t-test). (**F**) Comparative analysis of VP2 and VP5 release in HEK-293T cells expressing NS3/NS3A _WT_ or NS3/NS3A _N150Q_ mutant. Extracellular and intracellular fractions collected at 20 h post-transfection (h.p.t.) were analyzed by Western blot. (**G**) Quantification of extracellular VP2 and VP5 levels from panel F using ImageJ. Data represent mean ± SD (*n* = 3 biological replicates; **P < 0.01, ***P < 0.001; two-tailed unpaired t-test). (**H**) Dose-dependent release assay in the presence of NS3/NS3A variants. HEK-293T cells were co-transfected with VP2 or VP5 constructs and increasing amounts (0.05–5 μg) of NS3/NS3A (WT or N150Q) expression plasmids. (**I**) Quantification of extracellular VP2 and VP5 ratios from panel H using Image J (ns, not significant; *P < 0.05, ****P < 0.0001; two-way ANOVA with Šídák’s multiple-comparisons test).

Time-course analysis of viral protein expression further confirmed that loss of NS3/NS3A glycosylation reduced the release of extracellular viral particles in both MDOK and HEK-293T cells (Fig. 3A), consistent with the impaired release efficiency observed above (Fig. 2F and G). To directly visualize virion egress, MDOK cells were infected with BTV-20 _WT_ or BTV-20 _N150Q_ at an MOI of 10 for 12 h and analyzed by transmission electron microscopy (TEM). Ultrathin sections revealed numerous virions adjacent to the plasma membrane in BTV-20 _WT_-infected cells, frequently associated with membrane-budding profiles (Fig. 3B, upper panels), and quantification confirmed a higher number of budding events (Fig. 3C). In contrast, BTV-20 _N150Q_-infected cells displayed fewer membrane-associated particles and fewer discernible budding structures (Fig. 3B, lower panels; Fig. 3C). All these results indicated that NS3/NS3A glycosylation is critical for the late stages of the BTV life cycle, particularly for efficient virion trafficking and release.

Previous studies have shown that the release of VLPs composed of the BTV outer capsid proteins VP2 and VP5 depends on NS3/NS3A (26, 28, 29, 31, 32, 58, 60). We assumed that the glycosylation of NS3/NS3A might be required for the binding between NS3/NS3A and these outer capsid proteins to promote the release. Unexpectedly, Co-IP assays revealed that both the NS3/NS3A _WT_ and the NS3/NS3A _N150Q_ proteins bound VP2 and VP5 with comparable efficiency (Fig. 3D and E). In contrast, only NS3/NS3A _WT_ markedly enhanced the extracellular accumulation of VP2 and VP5 upon co-expression, whereas the N150Q mutant displayed a substantially reduced ability to promote their release (Fig. 3F and G). Although increasing the expression of NS3/NS3A _N150Q_ partially restored VP2 and VP5 release in a dose-dependent manner, its activity consistently remained lower than that of the WT NS3/NS3A protein (Fig. 3H and I). Together, these results indicate that loss of N-linked glycosylation at residue 150 reduces the ability of NS3/NS3A to promote capsid protein release, without affecting its direct binding to VP2 and VP5.

### NS3/NS3A glycosylation facilitates plasma membrane localization

**Fig 4:**
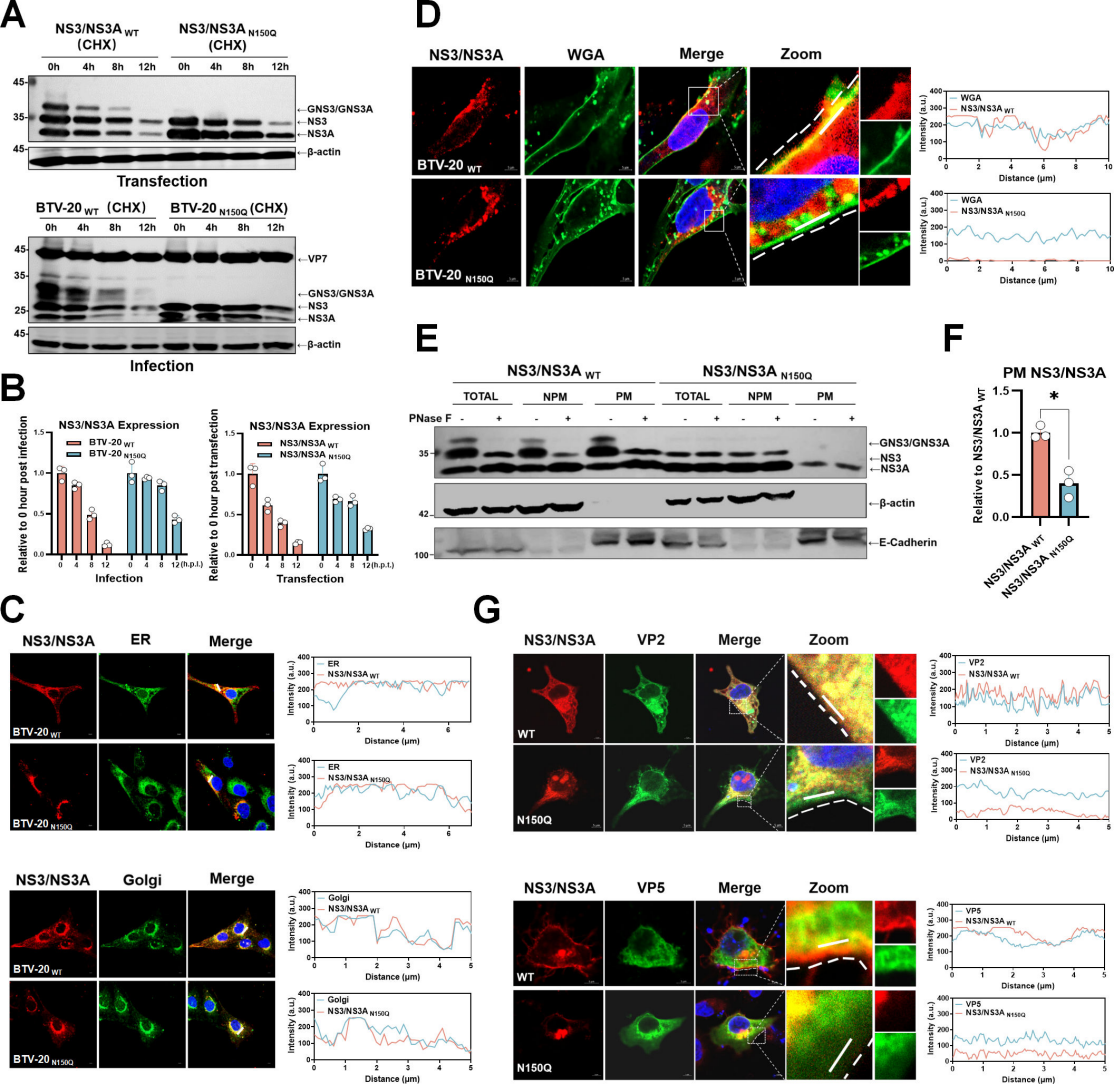
N-linked glycosylation drives plasma membrane accumulation of NS3/NS3A. (**A**) The stability of NS3/NS3A _WT_ and NS3/NS3A _N150Q_ proteins was examined in both transfected (top panels) and BTV-20-infected cells (bottom panels; MOI = 10). For transfection assays, HEK-293T cells were transfected with plasmids expressing NS3/NS3A _WT_ or the N150Q mutant and treated with cycloheximide (CHX; 100 μg/mL) at 18 h post-transfection (designated as 0 h post-CHX treatment) to block *de novo* protein synthesis. For infection assays, MDOK cells were infected with BTV-20 _WT_ or BTV-20 _N150Q_ and treated with CHX (100 μg/mL) at 10 h post-infection (designated as 0 h post-CHX treatment). Cells were harvested at the indicated time points and analyzed by Western blotting. (**B**) Quantification of NS3/NS3A protein levels shown in panel A was performed by ImageJ densitometric analysis. Protein levels at each time point were normalized to the corresponding 0 h post-CHX treatment (18 h post-transfection or 10 h post-infection, respectively). (**C**) Subcellular localization of NS3/NS3A in MDOK cells infected with BTV-20 _WT_ or BTV-20 _N150Q_ (MOI = 5, 12 h.p.i.). NS3/NS3A (red) was co-stained with ER marker anti-calnexin (green) or Golgi marker anti-syntaxin 6 (green). Fluorescence distribution was evaluated using line-scan intensity profiles. Scale bar, 5 µm. (**D**) Subcellular localization of NS3/NS3A in MDOK cells infected with BTV-20 _WT_ or BTV-20 _N150Q_ (MOI = 5, 12 h.p.i.). NS3/NS3A (red) was co-stained with plasma membrane marker WGA-Alexa Fluor 488 (green). Line-scan intensity profiles are shown. Scale bar, 5 µm. (**E**) Plasma membrane isolation of HEK-293T cells transfected with NS3/NS3A _WT_ or N150Q mutant, followed by Western blot analysis. PM (plasma membrane fraction); NPM (non-plasma membrane fraction); Total (plasma membrane fraction + non-plasma membrane fraction). (**F**) Quantification of NS3/NS3A at the plasma membrane fraction was analyzed by Image J from panel E (*P < 0.05, two-tailed unpaired t-test). (**G**) Confocal imaging of HeLa cells co-transfected with NS3/NS3A (WT or N150Q, red) and VP2 (green) or VP5 (green), showing their subcellular colocalization. Colocalization was assessed by line-scan intensity profiles.

Typically, N-linked glycans assist in protein folding, quality control, and ER-to-Golgi trafficking (48–52). To assess whether N-linked glycosylation affects NS3/NS3A stability and thereby contributes to its role in viral release, we performed cycloheximide (CHX) chase assays under both overexpression and infection conditions. These results showed that the N150Q mutation did not accelerate NS3/NS3A degradation, indicating that loss of glycosylation does not appreciably compromise NS3/NS3A stability (Fig. 4A and B). In addition, both NS3/NS3A_WT_ and the NS3/NS3A _N150Q_ mutant showed spatial colocalization with the markers of ER and Golgi apparatus, respectively (Fig. 4C; Fig. S3A and B). The ability of the NS3/NS3A _N150Q_ mutant to traffic to the Golgi, together with its degradation kinetics, suggests that N-linked glycosylation is not required for proper folding of NS3/NS3A under these conditions.

Interestingly, confocal microscopy revealed that glycosylation-competent NS3/NS3A preferentially accumulated at the plasma membrane, whereas the N150Q mutant exhibited reduced plasma membrane association in the context of both BTV-20 and BTV-1 infection (Fig. 4D; Fig. S3C), consistent with previous studies (36, 37). This observation was further validated by a plasma membrane isolation assay, which confirmed that PNGase F treatment completely removed the glycosylated forms of NS3/NS3A at the plasma membrane (Fig. 4E and F), indicating that N-linked glycosylation is critical for efficient localization of NS3/NS3A to the plasma membrane. Although both NS3/NS3A forms exhibited spatial proximity to the outer capsid proteins VP2 and VP5, the N150Q mutant displayed reduced surface-associated colocalization (Fig. 4G). Taken together, these results suggest that glycosylation at residue 150 is not essential for maintaining NS3/NS3A stability or its interaction with outer capsid proteins, but instead preferentially directs NS3/NS3A trafficking toward efficient plasma membrane localization.

### N-linked glycosylation promotes NS3/NS3A partitioning into lipid raft microdomains

**Fig 5:**
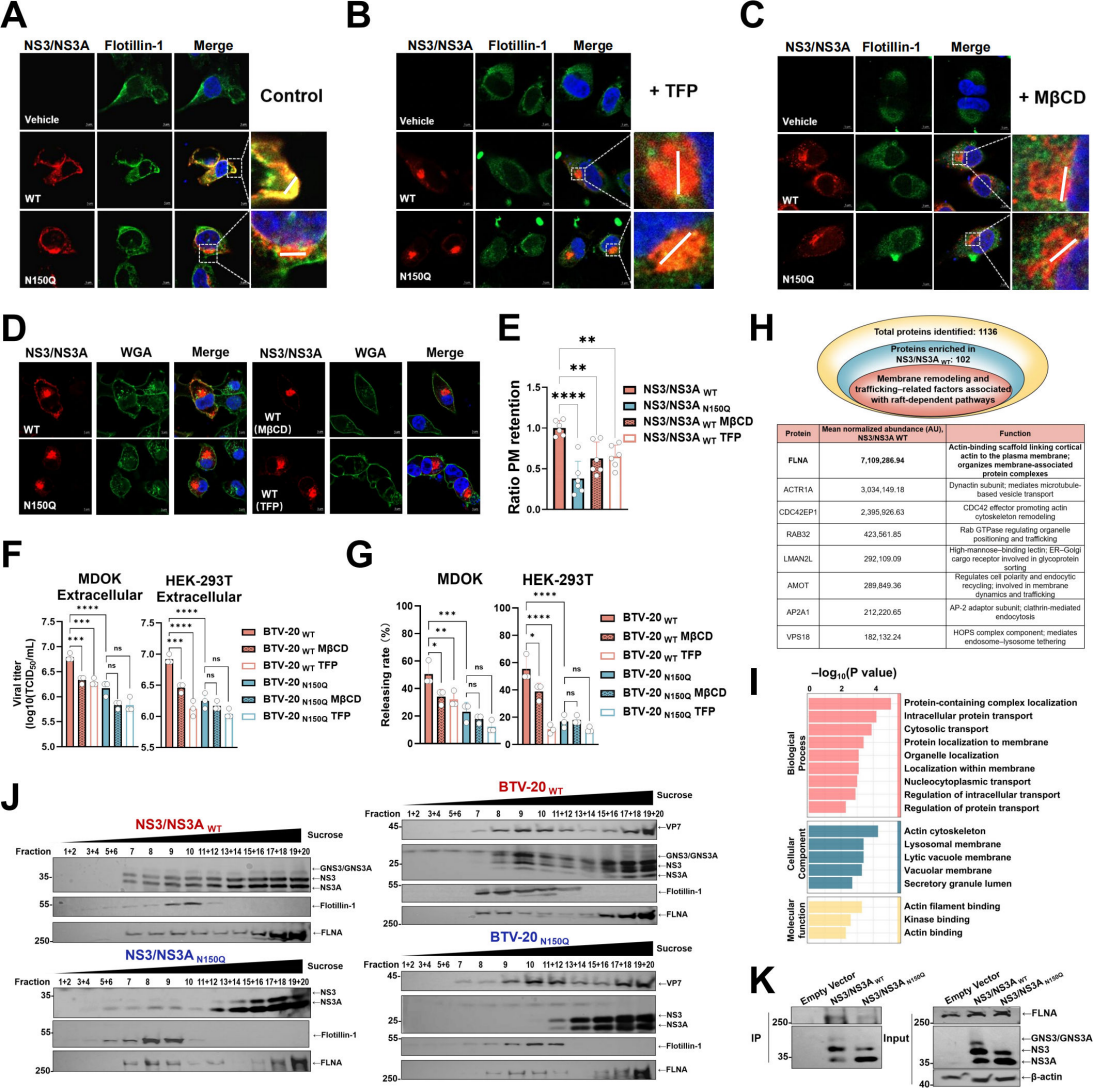
N-linked glycosylation of NS3/NS3A facilitates its raft-enriched membrane association and efficient BTV release. (**A**) Representative confocal images of HeLa cells transfected with NS3/NS3A _WT_ or the N150Q mutant. Cells were fixed and stained 20 h post-transfection. NS3/NS3A was detected using anti-NS3/NS3A (red), and lipid raft-enriched membrane domains were labeled with anti-flotillin-1 (green). Scale bars, 5 μm. (**B and C**) Confocal images of HeLa cells expressing NS3/NS3A treated with trifluoperazine (TFP) (**B**) or methyl-β-cyclodextrin (MβCD) (**C**). Cells were fixed and stained 20 h post-transfection. NS3/NS3A is shown in red and flotillin-1 in green. Fluorescence intensity profiles were analyzed along the indicated line scans. Scale bars, 5 μm. (**D**) Localization of NS3/NS3A at the plasma membrane of HeLa cells with or without MβCD or TFP treatment. The plasma membrane was stained using WGA-Alexa Fluor 488 (green), and NS3/NS3A was visualized in red. Scale bars, 5 μm. (**E**) Quantification of plasma membrane (PM) localization from panel D, shown as the ratio of PM-associated NS3/NS3A fluorescence intensity (defined by WGA staining) to total cellular NS3/NS3A intensity. Values were normalized to vehicle-treated controls and are presented as mean ± SD. Statistical analysis was performed using one-way ANOVA with Dunnett’s multiple comparisons test (**P < 0.01, ****P < 0.0001). (**F**) MDOK or HEK-293T cells were infected with BTV-20 _WT_ or BTV-20 _N150Q_ at an MOI of 5. At 6 h post-infection, the cells were treated with MβCD or TFP, and viral titers were determined at 12 h post-infection. Data represent mean ± SD (*n* = 3 biological replicates). Statistical significance was determined by two-way ANOVA with Šídák’s multiple comparisons test (ns, not significant; ***P < 0.001; ****P < 0.0001). (**G**) Analysis of BTV-20 release efficiency following raft disruption. MDOK cells were infected with BTV-20 (MOI = 5) and then treated with MβCD or TFP. Extracellular and intracellular viral titers were determined at the indicated time points. Release efficiency was calculated as [extracellular titer/(extracellular + intracellular titer)] × 100%. Data are presented as mean ± SD (*n* = 3 biological replicates). Statistical significance was determined using two-way ANOVA with Šídák’s multiple comparisons test (ns, not significant; *P < 0.05, **P < 0.01, ***P < 0.001, ****P < 0.0001). (**H**) A total of 1,136 host proteins were identified by quantitative proteomics, of which 102 proteins were enriched in the NS3/NS3A _WT_ interactome relative to the N150Q mutant (log₂ FC > 4 or unique to WT). Highlighted are the top eight candidates. (**I**) Gene Ontology (GO) enrichment analysis of proteins enriched in the NS3/NS3A _WT_ interactome. Significantly overrepresented terms are shown for the biological process, cellular component, and molecular function categories. Bars represent −log₁₀ (*P* value). (**J**) Sucrose gradient fractionation followed by immunoblotting of lysates from transfected HEK-293T cells, or from infected MDOK cells. DRM (raft-enriched) fractions correspond to fractions 5–12, whereas detergent-soluble membrane (DSM) fractions correspond to fractions 13–20. (**K**) Co-immunoprecipitation analysis of NS3/NS3A _WT_, NS3/NS3A _N150Q_, and FLNA. Cells were transfected with NS3/NS3A variants, followed by Co-IP.

Detergent-insoluble lipid raft microdomains, which are enriched in the plasma membrane, late secretory pathway, and endosomal compartments, serve as platforms for membrane-associated signaling and vesicle trafficking (61–64). Previous studies have reported that NS3/NS3A and progeny virions associate with raft-enriched structures (58, 65), raising the possibility that glycosylation may influence how NS3/NS3A engages these membrane environments during virus egress. To assess this, we examined colocalization of NS3/NS3A with flotillin-1-labeled lipid rafts. Confocal microscopy showed that NS3/NS3A _WT_ strongly associated with lipid rafts, whereas the glycosylation-deficient N150Q mutant displayed reduced colocalization under transfection conditions (Fig. 5A; Fig. S4B). Detergent-resistant membrane (DRM) fractionation further supported these observations: NS3/NS3A _WT_ was predominantly detected in raft fractions (fractions 5–12), whereas raft association of the N150Q mutant was significantly reduced under both transfection and infection conditions (Fig. 5J; Fig. S4F).

We next probed the glycan dependence of this association using the raft-disrupting agents methyl-β-cyclodextrin (MβCD) and trifluoperazine (TFP) (66). Both treatments reduced the colocalization of NS3/NS3A _WT_ with lipid rafts (Fig. 5B and C; Fig. S4B) and decreased its abundance at the plasma membrane (Fig. 5D and E). Functionally, disruption of lipid rafts impaired the release of infectious BTV-20 _WT_ and BTV-1 _WT_ particles, whereas the N150Q mutants, which already exhibit reduced raft association, were less sensitive to raft-disrupting treatments (Fig. 5F and G; Fig. S4C and D). This differential sensitivity indicates that the inhibitory effect of raft disruption on viral egress is at least partially dependent on NS3/NS3A glycosylation, supporting a model in which N-linked glycosylation enhances the ability of NS3/NS3A to partition into raft-enriched membrane domains that promote efficient virion release.

To identify host factors that may mediate this process, we performed AS-MS comparing NS3/NS3A _WT_ and the N150Q mutant. We identified 102 proteins that were enriched more than fourfold compared to the N150Q mutant or were unique to the NS3/NS3A _WT_ interactome (Fig. 5H). GO analysis revealed significant enrichment of pathways related to intracellular protein transport, membrane localization, and actin cytoskeleton organization (Fig. 5I). Among the enriched candidates, filamin A (FLNA), a scaffold protein that tethers and stabilizes lipid rafts by linking them to the actin cytoskeleton, was the most prominently enriched candidate (Fig. 5H) (67–69). Sucrose-gradient fractionation confirmed that FLNA partitions into detergent-insoluble raft fractions (Fig. 5K), and co-immunoprecipitation experiments further demonstrated that NS3/NS3A _WT_ associates with higher levels of FLNA than the N150Q mutant (Fig. 5L). Together, these results demonstrate that N-linked glycosylation of NS3/NS3A facilitates its recruitment into lipid raft microdomains, likely through specific interactions with raft-localized host proteins such as FLNA, thereby promoting efficient virion egress from infected cells.

### NS3 glycosylation enhances systemic viral dissemination and pathogenicity *in vivo*

**Fig 6:**
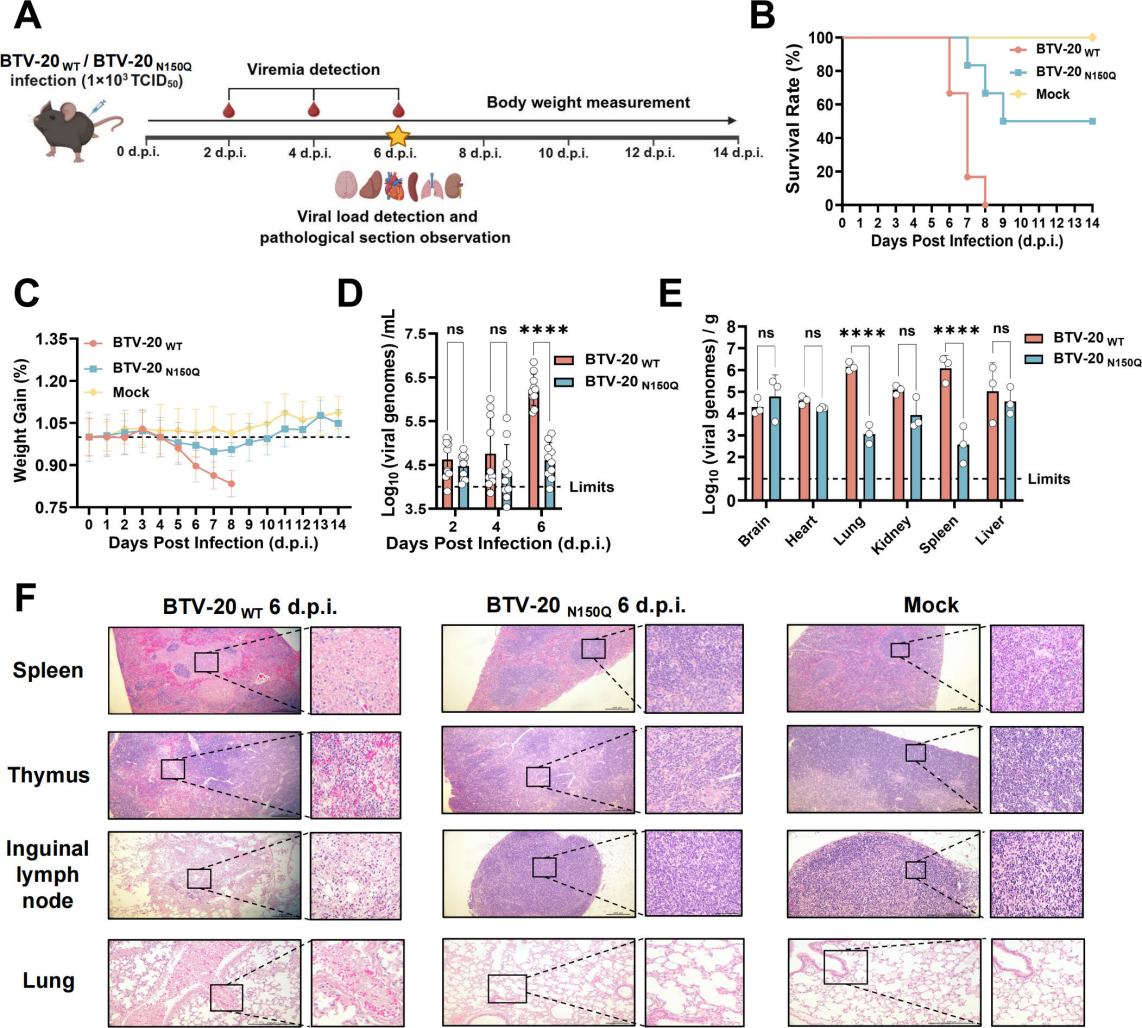
NS3/NS3A glycosylation promotes bluetongue virus pathogenicity in IFNAR-/- mice. (**A**) Schematic representation of the study design. IFNAR-/- mice (AG129) were subcutaneously inoculated with either BTV-20 _WT_ or the glycosylation-deficient mutant BTV-20 _N150Q_ (1 × 10³ TCID₅₀ per mouse). Schematic illustrations were generated using BioRender (https://www.biorender.com/library). (**B and C**) Survival curves (**B**) and body weight changes (**C**) were monitored daily for 14 days post-infection. Each group included six mice. (**D**) Viremia (whole blood) was quantified in individual mice at the indicated time points by RT-qPCR. Each group included 10 mice. Data were analyzed using two-way ANOVA with repeated measures, with Šídák’s test on log_10_-transformed data (ns, not significant; ****P < 0.0001). (**E**) Viral genome copies in various organs were quantified by RT-qPCR at 6 days post-infection. Each group included three mice. Data were analyzed using two-way ANOVA with Šídák’s multiple comparisons test on log₁₀-transformed data (ns, not significant; ****P < 0.0001). (**F**) Representative histopathological changes in the spleen, thymus, inguinal lymph nodes, and lung tissue from BTV-20 _WT_- or BTV-20 _N150Q_-infected mice. Hematoxylin and eosin (H&E) staining was performed at 6 days post-infection. Images from uninfected controls are shown for comparison. Scale bars, 200 μm.

Given that NS3/NS3A glycosylation enhances lipid-raft anchoring and virion release *in vitro*, we next examined whether this modification impacts viral dissemination and disease severity *in vivo* using the IFNAR-/- mice (AG129) model (Fig. 6A) (4, 70–75). AG129 mice were subcutaneously inoculated with either BTV-20 _WT_ or BTV-20 _N150Q_ at a dose of 10^3^ TCID_50_ per mouse. Striking differences in disease outcome were observed. WT-infected mice exhibited significantly accelerated mortality (Fig. 6B), more pronounced weight loss (Fig. 6C), and over 40-fold higher viremia (Fig. 6D) at 6 days post-infection compared to the BTV-20 _N150Q_ group. qRT-PCR analysis revealed markedly elevated viral genome loads in the lungs and spleens of BTV-20 _WT_-infected mice, with levels over 1,000-fold higher than those observed in BTV-20 _N150Q_-infected animals (Fig. 6E). These findings are consistent with our *in vitro* observations, supporting the role of NS3/NS3A glycosylation in enhancing viral dissemination. Histopathological analysis revealed severe lymphoid destruction in BTV-20 _WT_-infected mice, characterized by extensive necrosis of the splenic white pulp and marked lymphocyte depletion. Hemorrhage was observed in the thymus, along with lymphocyte necrosis and depletion in the inguinal lymph nodes. In the lungs, hemorrhage accompanied by fibrin deposition in small pulmonary veins was detected (Fig. 6F). In contrast, tissues from mice infected with the BTV-20 _N150Q_ mutant largely retained normal architecture. These results establish a clear link between NS3/NS3A glycosylation and enhanced systemic viral dissemination and tissue injury, underscoring the critical role of this post-translational modification in BTV pathogenesis.

## DISCUSSION

Although the glycosylation of NS3/NS3A has long been inferred, its precise glycan composition had not been defined. Here, we characterize the Asn_150_-linked glycan and show that it predominantly comprises high-mannose structures. Using a reverse-genetics approach, we further identify this modification as an important determinant of BTV egress and pathogenesis in mammalian hosts. Earlier work on related orbiviruses, such as the Ibaraki virus, suggested a potential role for NS3/NS3A glycosylation in virus release (76, 77), but these studies relied largely on broad glycosylation inhibitors and did not pinpoint the viral determinant. By introducing a site-specific N150Q mutation into two divergent BTV serotypes (BTV-20 and BTV-1), we demonstrate that the functional relevance of this glycan is conserved across lineages. More importantly, loss of glycosylation did not compromise NS3/NS3A stability or its interaction with outer capsid proteins; instead, it selectively biased NS3/NS3A toward the plasma membrane and altered its association with raft-enriched membranes. These observations suggest that the Asn_150_-linked glycan modulates the membrane trafficking and interaction profile of NS3/NS3A, positioning the protein within membrane microdomains that are permissive for viral egress. Consistent with this interpretation, pharmacological disruption of lipid rafts substantially reduced virus release, underscoring the functional contribution of raft-associated pools of NS3/NS3A during late stages of infection.

Proteomic analyses further revealed that NS3/NS3A glycosylation influences its access to membrane-associated trafficking and cytoskeletal factors. A subset of membrane-remodeling and scaffold-related proteins was significantly enriched in the WT-associated proteome, including factors linked to actin organization and membrane-cytoskeleton interfaces. Among these candidates, the actin scaffold protein FLNA emerged as a prominently enriched example. FLNA is known to contribute to the organization and stabilization of actin-supported membrane platforms and to the spatial coordination of protein complexes at specialized membrane sites (67, 68, 78). In the context of enveloped viruses such as HIV-1, FLNA has been shown to interact with viral structural proteins and to support productive particle assembly by stabilizing actin-supported membrane assembly sites, rather than by directly mediating membrane scission or budding (69). The preferential association of FLNA with glycosylated NS3/NS3A may therefore reflect glycan-dependent differences in how NS3/NS3A is positioned within raft-proximal membrane environments, rather than a direct role of FLNA in virion budding. Whether these glycan-dependent differences affect specific downstream trafficking routes or late-stage release steps will require further investigation.

The *in vivo* relevance of NS3/NS3A glycosylation was demonstrated in AG129 mice. Although these immunodeficient mice do not fully represent ruminant infection, they provide a practical system for assessing viral replication, dissemination, and NS3/NS3A-linked virulence. Importantly, our study focuses on a virus-intrinsic mechanism governing membrane trafficking and egress, rather than on host-specific immune responses, supporting the relevance of these findings across susceptible mammalian hosts. Glycosylation-deficient N150Q virus displayed markedly attenuated virulence, characterized by reduced viremia, limited tissue pathology, and restricted systemic spread. In contrast, BTV _WT_ caused extensive splenic necrosis, thymic hemorrhage, lymphoid depletion, and pulmonary vascular injury, highlighting the role of NS3/NS3A glycosylation in BTV pathogenicity. Given the high conservation of NS3/NS3A among BTV serotypes, including those infecting livestock, these glycan-dependent effects on viral egress and dissemination are likely to be relevant in natural ruminant hosts. Whether glycan shielding contributes to immune modulation in BTV, as observed in viruses such as HIV-1 and SARS-CoV-2 (46–48), remains an open question.

A notable feature of NS3/NS3A glycosylation is its host specificity. High-mannose glycans were readily detected in mammalian cells but largely absent in insect cells, consistent with minimal mobility changes following PNGase F treatment. This divergence may reflect an evolutionary trade-off in dual host adaptation: in vertebrate hosts, glycosylation enhances virulence and systemic dissemination, whereas in arthropod vectors, reduced glycosylation may promote persistence and limit cytopathology. The strict conservation of the Asn_150_ motif across all known BTV serotypes underscores its functional importance and highlights NS3/NS3A as a promising target for pan-serotype antiviral or vaccine strategies. Future studies evaluating glycosylation-deficient viruses in natural ruminant hosts will be essential for determining their immunogenicity and potential utility in vaccine development.

## Data Availability

All raw data supporting the findings of this study, including plaque assays, western blots, and qRT-PCR results, are available from the corresponding author upon reasonable request.

## SUPPLEMENTAL MATERIAL

Supplemental material jvi.02144-25-s0001.pdf Figures S1 to S4 and Tables S1 to S3.

ASM does not own the copyrights to Supplemental Material that may be linked to, or accessed through, an article. The authors have granted ASM a non-exclusive, world-wide license to publish the Supplemental Material files. Please contact the corresponding author directly for reuse.

## References

[1] Barua S, Rana EA, Prodhan MA, Akter SH, Gogoi-Tiwari J, Sarker S, Annandale H, Eagles D, Abraham S, Uddin JM. 2024. The global burden of emerging and re-emerging orbiviruses in livestock: an emphasis on bluetongue virus and epizootic hemorrhagic disease virus. Viruses 17:20. doi:10.3390/v17010020 39861809 PMC11768700

[2] Zhang S, Zhang Q, Zhang H, Liang R, Chen Q, Niu B. 2023. Assessing the export trade risk of bluetongue virus serotypes 4 and 8 in France. Risk Anal 43:1124–1136. doi:10.1111/risa.14011 35994609

[3] Thabet S, Lajnef R. 2024. Potential mechanisms underlying bluetongue virus emergence and spread. Front Virol 4. doi:10.3389/fviro.2024.1448192

[4] Schwartz-Cornil I, Mertens PPC, Contreras V, Hemati B, Pascale F, Bréard E, Mellor PS, MacLachlan NJ, Zientara S. 2008. Bluetongue virus: virology, pathogenesis and immunity. Vet Res 39:46. doi:10.1051/vetres:2008023 18495078

[5] Napp S, Gubbins S, Calistri P, Allepuz A, Alba A, García-Bocanegra I, Giovannini A, Casal J. 2011. Quantitative assessment of the probability of bluetongue virus overwintering by horizontal transmission: application to Germany. Vet Res 42:4. doi:10.1186/1297-9716-42-4 21314966 PMC3031226

[6] van Gennip RGP, van de Water SGP, van Rijn PA. 2014. Bluetongue virus nonstructural protein NS3/NS3a is not essential for virus replication. PLoS One 9:e85788. doi:10.1371/journal.pone.0085788 24465709 PMC3896414

[7] Courtejoie N, Bournez L, Zanella G, Durand B. 2019. Quantifying bluetongue vertical transmission in French cattle from surveillance data. Vet Res 50:34. doi:10.1186/s13567-019-0651-1 31088555 PMC6518818

[8] Stokstad E. 2024. Livestock virus hits Europe with a vengeance. Science 385:812–813. doi:10.1126/science.ads6026 39172842

[9] Barros SC, Maroco D, Henriques AM, Costa ML, Alves A, Ramos F, Duarte A, Fagulha T, Varanda IC, Santos FA dos, Ferreira AC, Barahona MJ, Carvalho PM, Orvalho M, Duarte MD. 2025. Fatal bluetongue virus serotype 3 infection in female dogs: a case report from Alentejo, Portugal, 2024. Viruses 17:159. doi:10.3390/v17020159 40006914 PMC11860487

[10] Carpi G, Holmes EC, Kitchen A. 2010. The evolutionary dynamics of bluetongue virus. J Mol Evol 70:583–592. doi:10.1007/s00239-010-9354-y 20526713

[11] Alkhamis MA, Aguilar-Vega C, Fountain-Jones NM, Lin K, Perez AM, Sánchez-Vizcaíno JM. 2020. Global emergence and evolutionary dynamics of bluetongue virus. Sci Rep 10:21677. doi:10.1038/s41598-020-78673-9 33303862 PMC7729867

[12] Stewart M, Hardy A, Barry G, Pinto RM, Caporale M, Melzi E, Hughes J, Taggart A, Janowicz A, Varela M, Ratinier M, Palmarini M. 2015. Characterization of a second open reading frame in genome segment 10 of bluetongue virus. J Gen Virol 96:3280–3293. doi:10.1099/jgv.0.000267 26290332 PMC4806581

[13] Belhouchet M, Mohd Jaafar F, Firth AE, Grimes JM, Mertens PPC, Attoui H. 2011. Detection of a fourth orbivirus non-structural protein. PLoS One 6:e25697. doi:10.1371/journal.pone.0025697 22022432 PMC3192121

[14] Ratinier M, Caporale M, Golder M, Franzoni G, Allan K, Nunes SF, Armezzani A, Bayoumy A, Rixon F, Shaw A, Palmarini M. 2011. Identification and characterization of a novel non-structural protein of bluetongue virus. PLoS Pathog 7:e1002477. doi:10.1371/journal.ppat.1002477 22241985 PMC3248566

[15] Mertens PP, Pedley S, Cowley J, Burroughs JN, Corteyn AH, Jeggo MH, Jennings DM, Gorman BM. 1989. Analysis of the roles of bluetongue virus outer capsid proteins VP2 and VP5 in determination of virus serotype. Virology (Auckl) 170:561–565. doi:10.1016/0042-6822(89)90447-9 2543130

[16] Fay PC, Attoui H, Batten C, Mohd Jaafar F, Lomonossoff GP, Daly JM, Mertens PPC. 2019. Bluetongue virus outer-capsid protein VP2 expressed in Nicotiana benthamiana raises neutralising antibodies and a protective immune response in IFNAR ^-/-^ mice. Vaccine X 2:100026. doi:10.1016/j.jvacx.2019.100026 31384743 PMC6668234

[17] Mohd Jaafar F, Belhouchet M, Vitour D, Adam M, Breard E, Zientara S, Mertens PPC, Attoui H. 2014. Immunisation with bacterial expressed VP2 and VP5 of bluetongue virus (BTV) protect α/β interferon-receptor knock-out (IFNAR(-/-)) mice from homologous lethal challenge. Vaccine (Auckl) 32:4059–4067. doi:10.1016/j.vaccine.2014.05.056 24886956

[18] Stewart M, Dovas CI, Chatzinasiou E, Athmaram TN, Papanastassopoulou M, Papadopoulos O, Roy P. 2012. Protective efficacy of Bluetongue virus-like and subvirus-like particles in sheep: presence of the serotype-specific VP2, independent of its geographic lineage, is essential for protection. Vaccine (Auckl) 30:2131–2139. doi:10.1016/j.vaccine.2012.01.042 22285887

[19] Janowicz A, Caporale M, Shaw A, Gulletta S, Di Gialleonardo L, Ratinier M, Palmarini M. 2015. Multiple genome segments determine virulence of bluetongue virus serotype 8. J Virol 89:5238–5249. doi:10.1128/JVI.00395-15 25822026 PMC4442542

[20] van Rijn PA, Maris-Veldhuis MA, van Gennip RGP. 2021. The bluetongue disabled infectious single animal (DISA) vaccine platform based on deletion NS3/NS3a protein is safe and protective in cattle and enables DIVA. Viruses 13:857. doi:10.3390/v13050857 34067226 PMC8151055

[21] Bonneau KR, Mullens BA, MacLachlan NJ. 2001. Occurrence of genetic drift and founder effect during quasispecies evolution of the VP2 and NS3/NS3A genes of bluetongue virus upon passage between sheep, cattle, and Culicoides sonorensis. J Virol 75:8298–8305. doi:10.1128/jvi.75.17.8298-8305.2001 11483775 PMC115074

[22] Feenstra F, Pap JS, van Rijn PA. 2015. Application of bluetongue disabled infectious single animal (DISA) vaccine for different serotypes by VP2 exchange or incorporation of chimeric VP2. Vaccine (Auckl) 33:812–818. doi:10.1016/j.vaccine.2014.12.003 25510389

[23] Feenstra F, van Gennip RGP, Maris-Veldhuis M, Verheij E, van Rijn PA. 2014. Bluetongue virus without NS3/NS3a expression is not virulent and protects against virulent bluetongue virus challenge. J Gen Virol 95:2019–2029. doi:10.1099/vir.0.065615-0 24914064

[24] Gould AR. 1988. Nucleotide sequence of the Australian bluetongue virus serotype 1 RNA segment 10. J Gen Virol 69 (Pt 4):945–949. doi:10.1099/0022-1317-69-4-945 2833571

[25] Feenstra F, van Gennip RGP, Schreuder M, van Rijn PA. 2016. Balance of RNA sequence requirement and NS3/NS3a expression of segment 10 of orbiviruses. J Gen Virol 97:411–421. doi:10.1099/jgv.0.000359 26644214

[26] Celma CCP, Roy P. 2009. A viral nonstructural protein regulates bluetongue virus trafficking and release. J Virol 83:6806–6816. doi:10.1128/JVI.00263-09 19369335 PMC2698550

[27] Celma CCP, Roy P. 2011. Interaction of calpactin light chain (S100A10/p11) and a viral NS protein is essential for intracellular trafficking of nonenveloped bluetongue virus. J Virol 85:4783–4791. doi:10.1128/JVI.02352-10 21411520 PMC3126219

[28] Labadie T, Jegouic S, Roy P. 2019. Bluetongue virus nonstructural protein 3 orchestrates virus maturation and drives non-lytic egress via two polybasic motifs. Viruses 11:1107. doi:10.3390/v11121107 31795485 PMC6949946

[29] Mohl BP, Kerviel A, Labadie T, Matsuo E, Roy P. 2020. Differential localization of structural and non-structural proteins during the bluetongue virus replication cycle. Viruses 12:343. doi:10.3390/v12030343 32245145 PMC7150864

[30] Hyatt AD, Zhao Y, Roy P. 1993. Release of bluetongue virus-like particles from insect cells is mediated by BTV nonstructural protein NS3/NS3A. Virology (Auckl) 193:592–603. doi:10.1006/viro.1993.1167 8384747

[31] Labadie T, Sullivan E, Roy P. 2020. Multiple routes of bluetongue virus egress. Microorganisms 8:965. doi:10.3390/microorganisms8070965 32605099 PMC7409164

[32] Roy P. 2020. Bluetongue virus assembly and exit pathways. Adv Virus Res 108:249–273. doi:10.1016/bs.aivir.2020.08.002 33837718 PMC7612823

[33] Rojas JM, Avia M, Martín V, Sevilla N. 2021. Inhibition of the IFN response by bluetongue virus: the story so far. Front Microbiol 12:692069. doi:10.3389/fmicb.2021.692069 34168637 PMC8217435

[34] Li Z, Lu D, Yang H, Li Z, Zhu P, Xie J, Liao D, Zheng Y, Li H. 2021. Bluetongue virus non-structural protein 3 (NS3) and NS4 coordinatively antagonize type Ⅰ interferon signaling by targeting STAT1. Vet Microbiol 254:108986. doi:10.1016/j.vetmic.2021.108986 33486325

[35] Pourcelot M, Zemirli N, Silva Da Costa L, Loyant R, Garcin D, Vitour D, Munitic I, Vazquez A, Arnoult D. 2016. The Golgi apparatus acts as a platform for TBK1 activation after viral RNA sensing. BMC Biol 14:69. doi:10.1186/s12915-016-0292-z 27538435 PMC4991008

[36] Wu X, Chen SY, Iwata H, Compans RW, Roy P. 1992. Multiple glycoproteins synthesized by the smallest RNA segment (S10) of bluetongue virus. J Virol 66:7104–7112. doi:10.1128/JVI.66.12.7104-7112.1992 1331513 PMC240390

[37] Bansal OB, Stokes A, Bansal A, Bishop D, Roy P. 1998. Membrane organization of bluetongue virus nonstructural glycoprotein NS3. J Virol 72:3362–3369. doi:10.1128/JVI.72.4.3362-3369.1998 9525663 PMC109819

[38] Labadie T, Roy P. 2020. A non-enveloped arbovirus released in lysosome-derived extracellular vesicles induces super-infection exclusion. PLoS Pathog 16:e1009015. doi:10.1371/journal.ppat.1009015 33075107 PMC7595637

[39] Hou X, Wang Y, Bu D, Wang Y, Sun S. 2023. EMNGly: predicting N-linked glycosylation sites using the language models for feature extraction. Bioinformatics 39:btad650. doi:10.1093/bioinformatics/btad650 37930896 PMC10627407

[40] Schwarz F, Aebi M. 2011. Mechanisms and principles of N-linked protein glycosylation. Curr Opin Struct Biol 21:576–582. doi:10.1016/j.sbi.2011.08.005 21978957

[41] Watanabe Y, Bowden TA, Wilson IA, Crispin M. 2019. Exploitation of glycosylation in enveloped virus pathobiology. Biochim Biophys Acta Gen Subj 1863:1480–1497. doi:10.1016/j.bbagen.2019.05.012 31121217 PMC6686077

[42] Evans DeWald L, Starr C, Butters T, Treston A, Warfield KL. 2020. Iminosugars: a host-targeted approach to combat Flaviviridae infections. Antiviral Res 184:104881. doi:10.1016/j.antiviral.2020.104881 32768411 PMC7405907

[43] Mathys L, François KO, Quandte M, Braakman I, Balzarini J. 2014. Deletion of the highly conserved N-glycan at Asn260 of HIV-1 gp120 affects folding and lysosomal degradation of gp120, and results in loss of viral infectivity. PLoS One 9:e101181. doi:10.1371/journal.pone.0101181 24967714 PMC4072736

[44] Kim JM, Yun SI, Song BH, Hahn YS, Lee CH, Oh HW, Lee YM. 2008. A single N-linked glycosylation site in the Japanese encephalitis virus prM protein is critical for cell type-specific prM protein biogenesis, virus particle release, and pathogenicity in mice. J Virol 82:7846–7862. doi:10.1128/JVI.00789-08 18524814 PMC2519568

[45] Hanna SL, Pierson TC, Sanchez MD, Ahmed AA, Murtadha MM, Doms RW. 2005. N-linked glycosylation of west nile virus envelope proteins influences particle assembly and infectivity. J Virol 79:13262–13274. doi:10.1128/JVI.79.21.13262-13274.2005 16227249 PMC1262570

[46] Vigerust DJ, Shepherd VL. 2007. Virus glycosylation: role in virulence and immune interactions. Trends Microbiol 15:211–218. doi:10.1016/j.tim.2007.03.003 17398101 PMC7127133

[47] Zhang F, Schmidt F, Muecksch F, Wang Z, Gazumyan A, Nussenzweig MC, Gaebler C, Caskey M, Hatziioannou T, Bieniasz PD. 2024. SARS-CoV-2 spike glycosylation affects function and neutralization sensitivity. mBio 15:e0167223. doi:10.1128/mbio.01672-23 38193662 PMC10865855

[48] Shajahan A, Pepi LE, Rouhani DS, Heiss C, Azadi P. 2021. Glycosylation of SARS-CoV-2: structural and functional insights. Anal Bioanal Chem 413:7179–7193. doi:10.1007/s00216-021-03499-x 34235568 PMC8262766

[49] Feng T, Zhang J, Chen Z, Pan W, Chen Z, Yan Y, Dai J. 2022. Glycosylation of viral proteins: implication in virus-host interaction and virulence. Virulence 13:670–683. doi:10.1080/21505594.2022.2060464 35436420 PMC9037552

[50] Ishida K, Yagi H, Kato Y, Morita E. 2023. N-linked glycosylation of flavivirus E protein contributes to viral particle formation. PLoS Pathog 19:e1011681. doi:10.1371/journal.ppat.1011681 37819933 PMC10593244

[51] Nurdin JA, Kotaki T, Kawagishi T, Sato S, Yamasaki M, Nouda R, Minami S, Kanai Y, Kobayashi T. 2023. N-glycosylation of rotavirus NSP4 protein affects viral replication and pathogenesis. J Virol 97:e0186122. doi:10.1128/jvi.01861-22 36598201 PMC9888287

[52] Yap SSL, Nguyen-Khuong T, Rudd PM, Alonso S. 2017. Dengue virus glycosylation: what do we know? Front Microbiol 8:1415. doi:10.3389/fmicb.2017.01415 28791003 PMC5524768

[53] Qi Y, Wang F, Chang J, Jiang Z, Sun C, Lin J, Wu J, Yu L. 2022. Genetic characteristics and pathogenicity of the first bluetongue virus serotype 20 strain isolated in China. Transbounding Emerging Dis 69:e2164–e2174. doi:10.1111/tbed.14555 35403352

[54] Boyce M, Celma CCP, Roy P. 2008. Development of reverse genetics systems for bluetongue virus: recovery of infectious virus from synthetrantic RNA transcripts. J Virol 82:8339–8348. doi:10.1128/JVI.00808-08 18562540 PMC2519640

[55] Pretorius JM, Huismans H, Theron J. 2015. Establishment of an entirely plasmid-based reverse genetics system for bluetongue virus. Virology (Auckl) 486:71–77. doi:10.1016/j.virol.2015.09.004 26408855

[56] Liang J, Wan Y, Gao J, Zheng L, Wang J, Wu P, Li Y, Wang B, Wang D, Ma Y, et al.. 2024. Erythroid-intrinsic activation of TLR8 impairs erythropoiesis in inherited anemia. Nat Commun 15. doi:10.1038/s41467-024-50066-w PMC11227506 38971858

[57] Schöl M, Schempp R, Hennig T, Wigger D, Schumacher F, Kleuser B, Stigloher C, van Ham M, Jänsch L, Schneider-Schaulies S, Dölken L, Avota E. 2024. Dynamic changes in the proximitome of neutral sphingomyelinase-2 (nSMase2) in TNFα stimulated Jurkat cells. Front Immunol 15:1435701. doi:10.3389/fimmu.2024.1435701 39044828 PMC11263205

[58] Bhattacharya B, Roy P. 2008. Bluetongue virus outer capsid protein VP5 interacts with membrane lipid rafts via a SNARE domain. J Virol 82:10600–10612. doi:10.1128/JVI.01274-08 18753209 PMC2573168

[59] Amiar S, Johnson KA, Husby ML, Marzi A, Stahelin RV. 2024. A fatty acid-ordered plasma membrane environment is critical for Ebola virus matrix protein assembly and budding. J Lipid Res 65:100663. doi:10.1016/j.jlr.2024.100663 39369791 PMC11565396

[60] Beaton AR, Rodriguez J, Reddy YK, Roy P. 2002. The membrane trafficking protein calpactin forms a complex with bluetongue virus protein NS3 and mediates virus release. Proc Natl Acad Sci USA 99:13154–13159. doi:10.1073/pnas.192432299 12235365 PMC130602

[61] Salaün C, James DJ, Chamberlain LH. 2004. Lipid rafts and the regulation of exocytosis. Traffic 5:255–264. doi:10.1111/j.1600-0854.2004.0162.x 15030567 PMC2394575

[62] Simons K, Sampaio JL. 2011. Membrane organization and lipid rafts. Cold Spring Harb Perspect Biol 3:a004697. doi:10.1101/cshperspect.a004697 21628426 PMC3179338

[63] Levental I, Levental KR, Heberle FA. 2020. Lipid rafts: controversies resolved, mysteries remain. Trends Cell Biol 30:341–353. doi:10.1016/j.tcb.2020.01.009 32302547 PMC7798360

[64] Rajendran L, Simons K. 2005. Lipid rafts and membrane dynamics. J Cell Sci 118:1099–1102. doi:10.1242/jcs.01681 15764592

[65] Bhattacharya B, Roy P. 2010. Role of lipids on entry and exit of bluetongue virus, a complex non-enveloped virus. Viruses 2:1218–1235. doi:10.3390/v2051218 21994677 PMC3187602

[66] Simons K, Ikonen E. 1997. Functional rafts in cell membranes. Nature 387:569–572. doi:10.1038/42408 9177342

[67] Tavano R, Contento RL, Baranda SJ, Soligo M, Tuosto L, Manes S, Viola A. 2006. CD28 interaction with filamin-A controls lipid raft accumulation at the T-cell immunological synapse. Nat Cell Biol 8:1270–1276. doi:10.1038/ncb1492 17060905

[68] Sverdlov M, Shinin V, Place AT, Castellon M, Minshall RD. 2009. Filamin A regulates caveolae internalization and trafficking in endothelial cells. Mol Biol Cell 20:4531–4540. doi:10.1091/mbc.e08-10-0997 19759182 PMC2770941

[69] Cooper J, Liu L, Woodruff EA, Taylor HE, Goodwin JS, D’Aquila RT, Spearman P, Hildreth JEK, Dong X. 2011. Filamin A protein interacts with human immunodeficiency virus type 1 Gag protein and contributes to productive particle assembly. J Biol Chem 286:28498–28510. doi:10.1074/jbc.M111.239053 21705339 PMC3151092

[70] Saminathan M, Singh KP, Vineetha S, Maity M, Biswas SK, Manjunathareddy GB, Chauhan HC, Milton AAP, Ramakrishnan MA, Maan S, Maan NS, Hemadri D, Chandel BS, Gupta VK, Mertens PPC. 2020. Virological, immunological and pathological findings of transplacentally transmitted bluetongue virus serotype 1 in IFNAR1-blocked mice during early and mid gestation. Sci Rep 10:2164. doi:10.1038/s41598-020-58268-0 32034180 PMC7005837

[71] Marín-Lopez A, Calvo-Pinilla E, Moreno S, Utrilla-Trigo S, Nogales A, Brun A, Fikrig E, Ortego J. 2019. Modeling arboviral infection in mice lacking the interferon alpha/beta receptor. Viruses 11:35. doi:10.3390/v11010035 30625992 PMC6356211

[72] Saminathan M, Singh KP, Maity M, Vineetha S, Manjunathareddy GB, Dhama K, Malik YS, Ramakrishnan MA, Misri J, Gupta VK. 2021. Pathological and immunological characterization of bluetongue virus serotype 1 infection in type I interferons blocked immunocompetent adult mice. J Adv Res 31:137–153. doi:10.1016/j.jare.2021.01.007 34194838 PMC8240118

[73] Calvo-Pinilla E, Rodríguez-Calvo T, Anguita J, Sevilla N, Ortego J. 2009. Establishment of a bluetongue virus infection model in mice that are deficient in the alpha/beta interferon receptor. PLoS One 4:e5171. doi:10.1371/journal.pone.0005171 19357779 PMC2663843

[74] Coetzee P, van Vuuren M, Venter E, Stokstad M. 2014. A review of experimental infections with bluetongue virus in the mammalian host. Virus Res 182:21–34. doi:10.1016/j.virusres.2013.12.044 24462840 PMC7132480

[75] Maclachlan NJ. 2011. Bluetongue: history, global epidemiology, and pathogenesis. Prev Vet Med 102:107–111. doi:10.1016/j.prevetmed.2011.04.005 21570141

[76] Urata M, Watanabe R, Iwata H. 2014. The host specific NS3 glycosylation pattern reflects the virulence of Ibaraki virus in different hosts. Virus Res 181:6–10. doi:10.1016/j.virusres.2013.12.027 24389093

[77] Urata M, Watanabe R, Iwata H. 2015. The effect of glycosylation on cytotoxicity of Ibaraki virus nonstructural protein NS3. J Vet Med Sci 77:1611–1616. doi:10.1292/jvms.15-0121 26178820 PMC4710717

[78] Mammoto A, Huang S, Ingber DE. 2007. Filamin links cell shape and cytoskeletal structure to Rho regulation by controlling accumulation of p190RhoGAP in lipid rafts. J Cell Sci 120:456–467. doi:10.1242/jcs.03353 17227794

