# Supplementary material for: Glycosylated NS3/NS3A protein of bluetongue virus facilitates efficient viral egress via lipid raft anchoring
Source: J Virol. 2026 Feb 18;100(3):e02144-25. doi: 10.1128/jvi.02144-25 (PMC13011428; doi:10.1128/jvi.02144-25)
Supplement: Supplemental material — Figures S1 to S4 and Tables S1 to S3. [file jvi.02144-25-s0001.pdf]

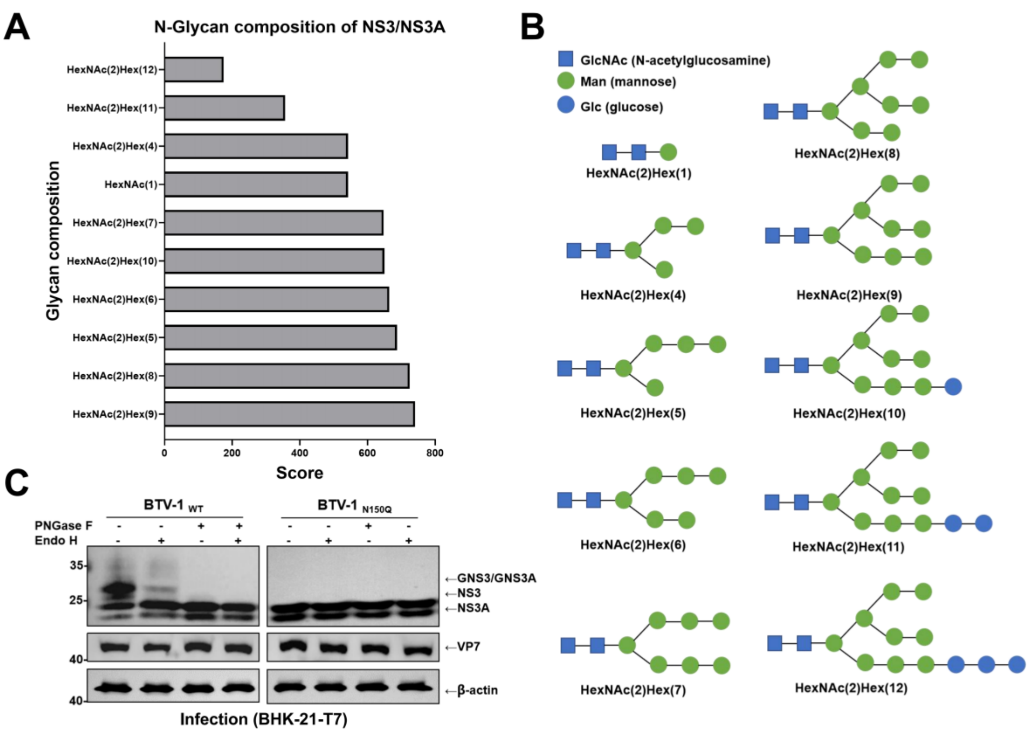

Supplementary Figure S1. Characterization of the N-glycan composition and glycosylation status of NS3/NS3A. (A) N-Glycan composition associated with the NS3/NS3A glycoprotein. This analysis was performed using the NS3/NS3A proteins expressed in HEK-293T cells. Bar plot showing the spectral-matching scores for the major N-glycan species identified on GNS3/NS3A. Scores represent algorithm-generated measures of MS/MS matching quality, reflecting the confidence supporting each glycan composition. All identified glycans share the conserved di-GlcNAc core ($HexNAc_2$) and differ primarily in mannose content (Hex_{1-12}), consistent with high-mannose-type structures. **(B)** Structural representations of the predominant N-glycan species detected on GNS3/ NS3A. Blue squares indicate GlcNAc, green circles mannose, and blue circles glucose. **(C)** Validation of NS3/NS3A glycosylation in BTV-1 infected BHK-21-T7 cells (MOI = 10, 24 hpi). Cell lysates from BTV-1 $_{WT}$ or BTV-1 $_{N150Q}$ infected cells were treated with PNGase F or Endo H and analyzed by immunoblotting to assess glycan sensitivity and confirm loss of glycosylation in the N150Q mutant.

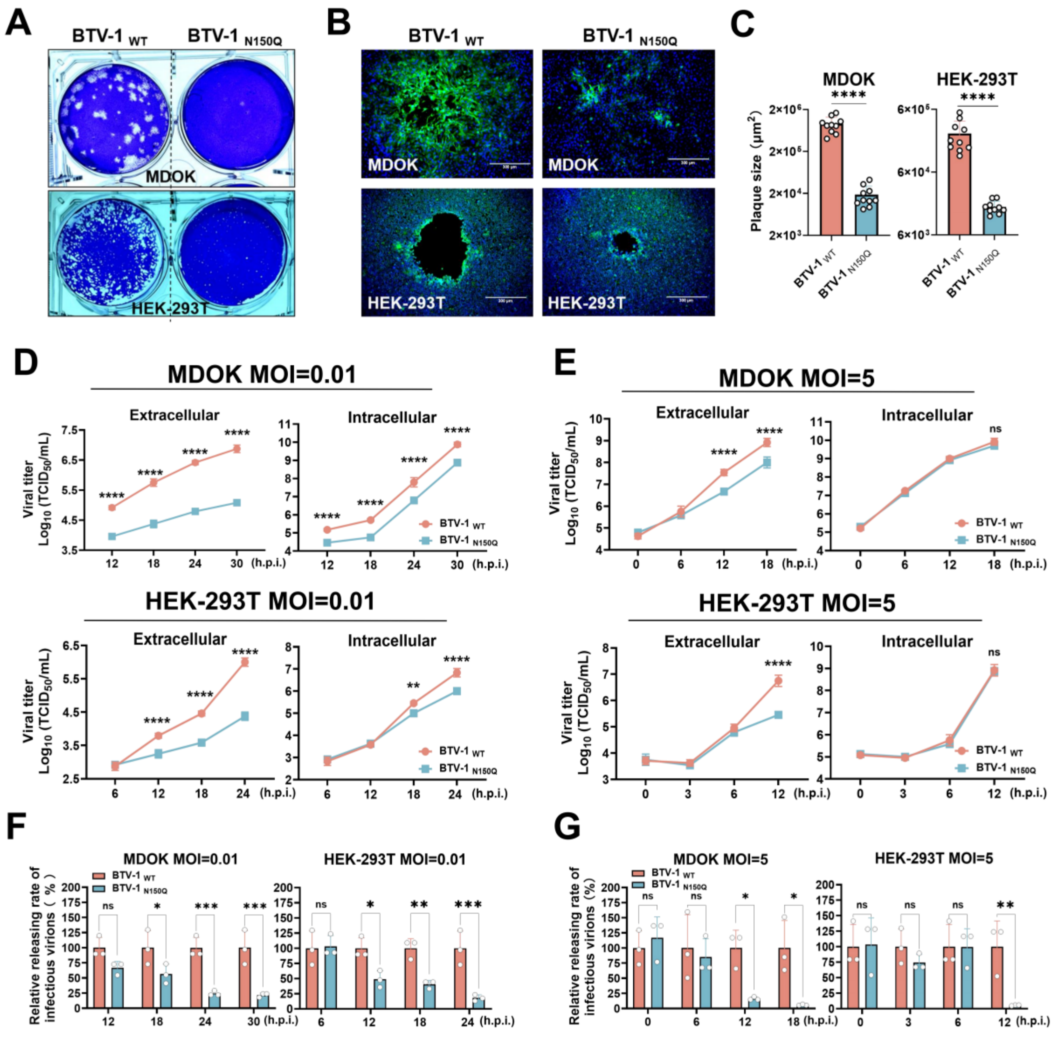

Supplementary Figure S2. Loss of N-linked glycosylation of NS3/NS3A reduces BTV-1 infective virions release

(A) Plaques in MDOK and HEK-293T cells infected with either BTV-1$_{\text{WT}}$ or BTV-1$_{\text{N150Q}}$. Monolayers were infected with BTV-1 variants and overlaid with low-melting-point agarose. Plaques were visualized by crystal violet staining at 7 d.p.i. (MDOK) or 3 d.p.i. (HEK-293T). **(B)** Immunofluorescence-based plaque assay. Infected cells were overlaid with methylcellulose, fixed at 7 d.p.i. (MDOK) or 5 d.p.i. (HEK-293T), and stained with an anti-VP7 monoclonal antibody followed by an Alexa Fluor™ 488-conjugated secondary antibody. Scale bar, 300 μm. **(C)** Quantification of plaque areas (n = 10 plaques per virus) from panel **B** using ImageJ. Data are presented as mean ± SD (two-tailed unpaired t-test; ****$p < 0.0001$). **(D–E)** Multicycle (MOI = 0.01; **D**) and single-cycle (MOI = 5; **E**) growth kinetics of BTV-1$_{\text{WT}}$ and BTV-1$_{\text{N150Q}}$ in MDOK and HEK-293T cells. Both of extracellular and intracellular

infectious titers were quantified by $TCID_{50}$ assay. Data were log_{10}-transformed and are presented as mean ± SD (n = 3 biological replicates; two-way ANOVA with Šídák's multiple-comparison test; *$p < 0.05$, **$p < 0.01$, ***$p < 0.001$, ****$p < 0.0001$). **(F-G)** Relative releasing efficiency was calculated as [extracellular / (extracellular + intracellular)] for each condition. Values were normalized to the corresponding WT at each time point (set to 1) to allow comparison across different conditions. Bars represent mean ± SD (n = 3 biological replicates; ns, not significant; *$p < 0.05$, **$p < 0.01$, ***$p < 0.001$, ****$p < 0.0001$; two-way ANOVA with Šídák's test).

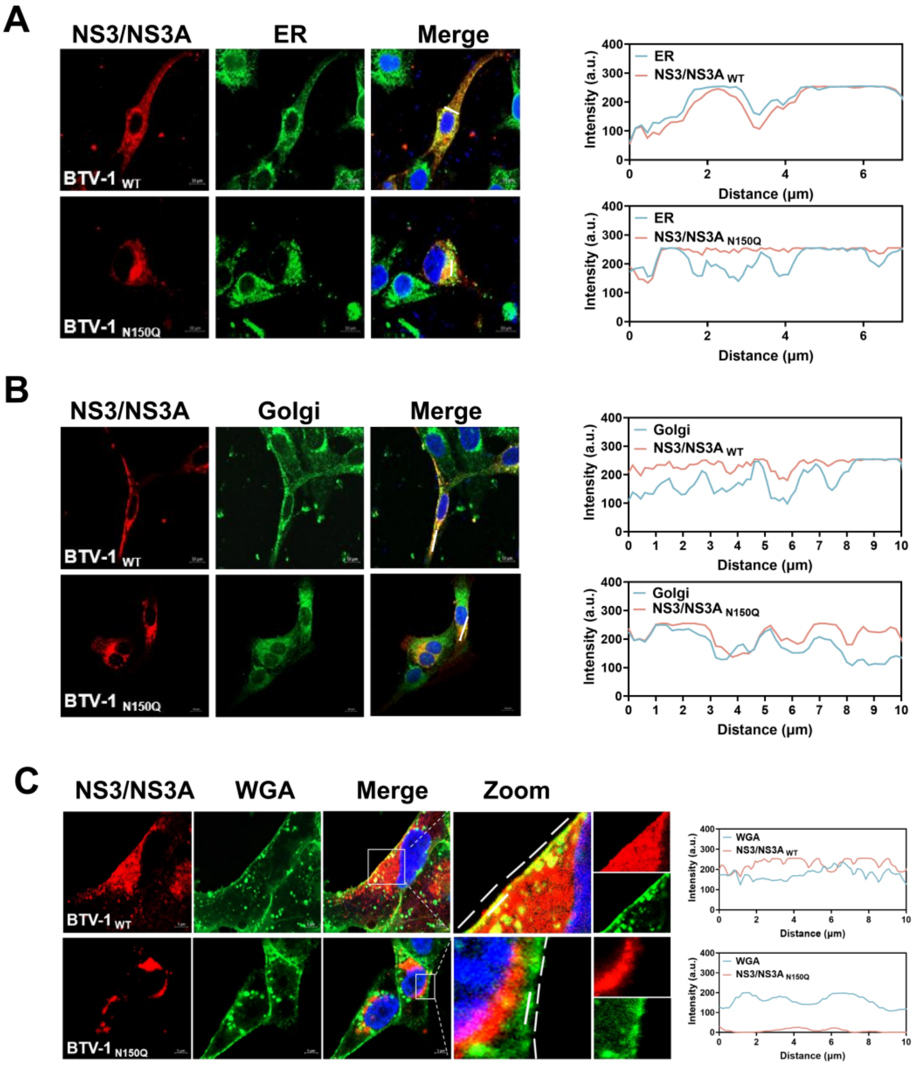

Supplementary Figure S3. N-linked glycosylation drives NS3/NS3A accumulation at plasma membrane. Subcellular localization of NS3/NS3A in MDOK cells infected with BTV-1 $_{WT}$ or BTV-1 $_{N150Q}$ (MOI = 5, 12 h.p.i.). NS3/NS3A (red) was co-stained with **(A)** ER marker anti-calnexin (green) or **(B)** Golgi marker anti-syntaxin 6 (green). Fluorescence distribution was evaluated using line-scan intensity profiles. Scale bar, 10 μm. **(C)** Subcellular localization of NS3/NS3A in MDOK cells infected with BTV-1 $_{WT}$ or BTV-1 $_{N150Q}$ (MOI = 5, 12 h.p.i.). NS3/NS3A (red) was co-stained with plasma membrane marker WGA-Alexa Fluor 488 (green). Line-scan intensity profiles are shown. Scale bar, 5 μm.

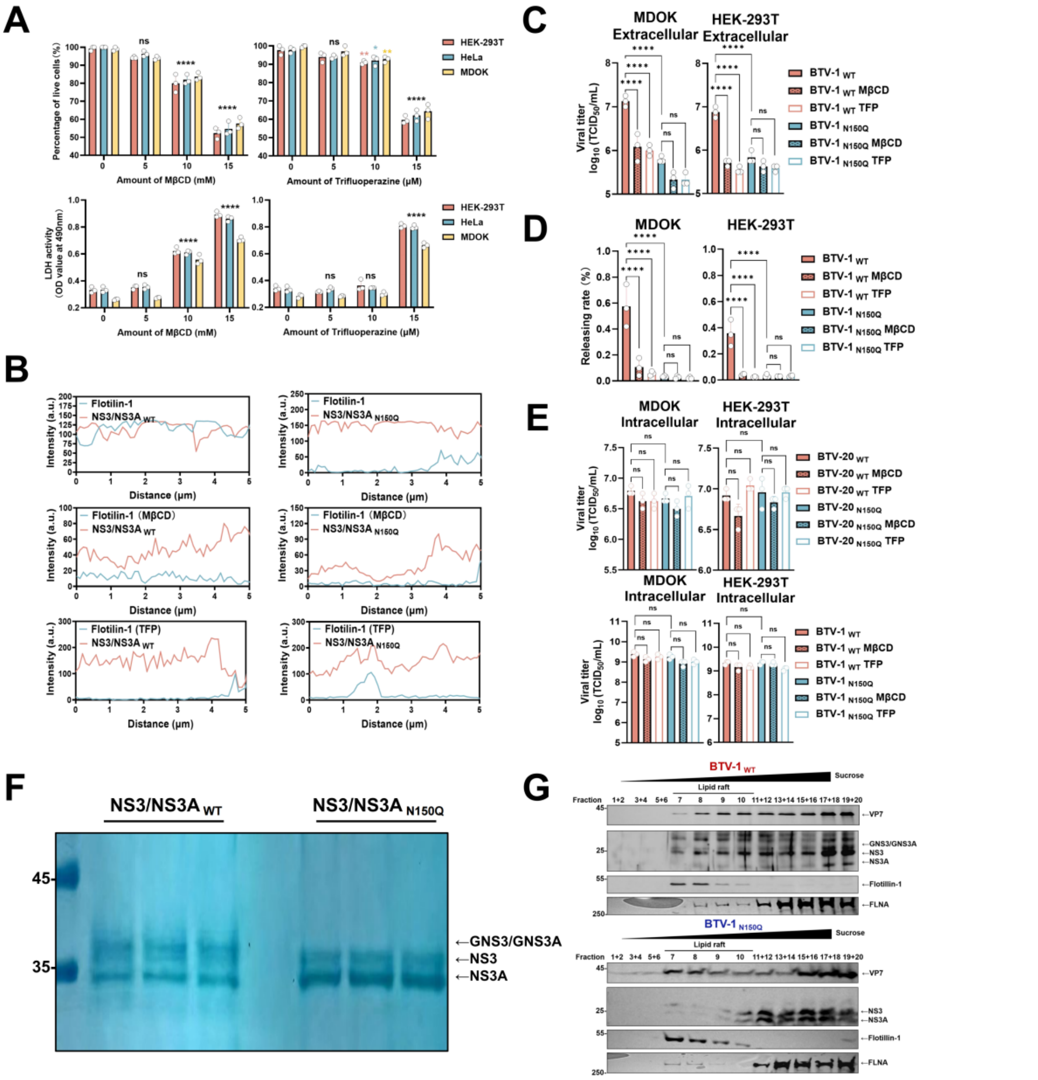

Supplementary Fig. 4 N-linked glycosylation of NS3/NS3A facilitates its raft-enriched membrane association and efficient BTV release (A) Cell viability analysis. Cell viability was assessed by Trypan blue exclusion, and cytotoxicity was measured by LDH release following treatment with increasing concentrations of methyl-β-cyclodextrin (MβCD) or trifluoperazine (TFP). Statistical significance was assessed by one-way ANOVA followed by Dunnett's multiple comparisons test. For each cell line, all treatment conditions were compared only with the corresponding vehicle control within the same cell line. Black asterisks (or "ns") indicate statistical significance relative to the control. ns, not significant; *$p < 0.05$; **$p < 0.01$; ***$p < 0.001$; ****$p < 0.0001$. **(B)** Fluorescence intensity profiles corresponding to **Fig. 5A-C** were analyzed along the indicated line scans to evaluate the spatial relationship between NS3/NS3A and flotillin-1 under the indicated conditions. **(C)** MDOK or HEK-293T cells were

infected with BTV-1$_{WT}$ or BTV-1 $_{N150Q}$ at a MOI of 5, treated with MβCD or TFP at 6 h postinfection. Viral titers were determined at 12 h postinfection. Data represent mean ± SD (n = 3 biological replicates). Statistical significance was determined by two-way ANOVA with Šídák's multiple comparisons test (****$p < 0.0001$). **(D)** Analysis of BTV-1 release efficiency following raft disruption. MDOK cells were infected with BTV-1 (MOI = 5) and treated with MβCD or TFP. Extracellular and intracellular viral titers were determined, and release efficiency was calculated as [extracellular titer / (extracellular + intracellular titer)] × 100%. Data are presented as mean ± SD (n = 3 biological replicates). Statistical significance was determined using two-way ANOVA with Šídák's multiple comparisons test (*$p < 0.05$, **$p < 0.01$, ***$p < 0.001$, ****$p < 0.0001$). **(E)** MDOK or HEK-293T cells were infected with viruses at a MOI of 5, treated with MβCD or TFP at 6 h postinfection, and intracellular viral titers were determined at 12 h postinfection. Data represent mean ± SD (n = 3 biological replicates). Statistical significance was determined by two-way ANOVA with Šídák's multiple comparisons test (ns, not significant). **(F)** SDS-PAGE analysis of affinity-purified NS3/NS3A $_{WT}$ and NS3/NS3A $_{N150Q}$ proteins expressed in HEK-293T cells. Proteins were purified using anti-FLAG affinity beads and subsequently used as input material for mass spectrometry-based proteomic analysis. **(G)** Sucrose gradient fractionation followed by immunoblotting of lysates from MDOK cells infected with BTV-1 $_{WT}$ or BTV-1 $_{N150Q}$. Detergent-resistant membrane (DRM; raft-enriched) fractions correspond to fractions 5-12, whereas detergent-soluble membrane (DSM) fractions correspond to fractions 13-20.

Table S1. GenBank accession numbers of NS3/NS3A amino acid sequences from Bluetongue virus (BTV) serotypes included in the multiple sequence alignment

BTV serotype	Strain	Genbank Accession Number
1	RSArrrr/01	JX680466
2	RSArrrr/2	KP821995.1
3	RSArrrr/03	KP822004.1
4	RSArrrr/04	KP822031.1
5	RSArrrr/05	MN710354.1
6	RSArrrr/06	GQ506505.1
7	RSArrrr/07	MN710355.1
8	RSArrrr/08	KP822041.1
9	RSArrrr/09	KP822053.1
10	RSArrrr/10	MN710356.1
11	RSArrrr/11	MN710357.1
12	RSArrrr/12	MN710358.1
13	RSArrrr/13	MN710359.1
14	RSArrrr/14	KP821948.1
15	RSArrrr/15	KP821949.1
16	RSArrrr/16	JX129386.1
17	RSArrrr/17	MN710360.1
18	USA2014	KX164128.1

19	USA2003	KF986511.1
20	GX015	OL333543.1
21	5149E	MG206086.1
22	USA2005	KX164148.1
23	IND1988	KP696525.1
24	USA2007	KX164158.1

Table S2. List of plasmids constructed and used in this study

Recombinant DNA	Resource
pCAGGS-VP2$_{BTV-20}$-2×HA	This study
pCAGGS-VP5$_{BTV-20}$-2×HA	This study
pCAGGS-NS3/NS3A$_{BTV-20/WT}$-3×Flag-2×HA	This study
pCAGGS-NS3/NS3A$_{BTV-20/N50Q}$-3×Flag-2×HA	This study
pCAGGS-NS3/NS3A$_{BTV-20/WT}$	This study
pCAGGS-NS3/NS3A$_{BTV-20/N50Q}$	This study

Table S3. Primers and probes used in viral RNA quantification

Primers name	Primers sequence (5'-3')	Probe	Target genes
BTV-Fwd	TGGATAAAGCGATGTCAAA	FAM-AAGCTGCATTCGCATCGTACGC-BHQ	BTV-20 Segment 10
BTV-Rev	ACATCATCACGAAACGCTTC		
